# Clinical, social, and economic burdens of schizophrenia in Japan: a targeted literature review

**DOI:** 10.1038/s41537-025-00716-9

**Published:** 2026-01-20

**Authors:** Fumiko Ono, Miyu Okamura

**Affiliations:** 1 Nippon Boehringer Ingelheim Co., Ltd., Tokyo, Japan; 2 Syneos Health Japan K.K., Tokyo, Japan

**Keywords:** Diseases, Psychiatric disorders, Schizophrenia

## Abstract

Schizophrenia is a severe mental disorder with substantial clinical, economic, and humanistic impacts. This targeted literature review evaluated the burden of schizophrenia on patients and caregivers in Japan. Data were collected from PubMed, Ichushi, CiNii, J-STAGE, and the Cochrane Database (2013–2023) and supplementary materials from medical associations, government agencies, and patient organizations (2018–2023). The review focused on epidemiology, clinical management, societal, humanistic, and economic burdens experienced by patients and caregivers. The review identified 156 journal publications, 73 conference proceedings, and 37 additional data sources. Obesity, depression, and type 2 diabetes were highlighted as frequent comorbidities. Cognitive impairment in schizophrenia, assessed by the Brief Assessment of Cognition in Schizophrenia, indicated severe functional deficits with a Z-score of -2.1. Issues related to long-term hospitalization, including social isolation and inadequate post-discharge support, were also reported. Interventions aimed at improving cognitive function, fostering self-care, and strengthening community cooperation were identified as key factors in reducing early readmission rates. Caregivers experienced significant productivity losses, particularly due to presenteeism, leading to an estimated annual loss of JPY 2.4 million. The hand search further revealed a lack of stakeholder-driven initiatives to address the comprehensive burdens of schizophrenia, such as awareness campaigns, educational programs, and multidisciplinary approaches. This review underscores the multifaceted burdens of schizophrenia in Japan, emphasizing the urgent need for coordinated, evidence-based countermeasures involving multiple stakeholders, including patients, caregivers, healthcare professionals, and policymakers. To reduce burdens and improve healthcare, further research is needed to bridge the gap between required interventions and stakeholder engagement.

## Introduction

Schizophrenia is a chronic and severe mental disorder that profoundly affects an individual’s thinking, emotions, and behavior. Globally, approximately 23 million people are estimated to live with schizophrenia^1^, with a lifetime prevalence of approximately 0.3–0.7%^2,3^. This disorder significantly disrupts patients’ lives, creating challenges in interpersonal relationships and employment^4^. These difficulties impose substantial social burdens on patients, including stigma and social exclusion, which may hinder reintegration into society and contribute to increasing unemployment and long-term disability.

In Japan, approximately 0.6% of the population is affected by schizophrenia^5^. The healthcare system is based on universal public insurance, which covers mental healthcare, including outpatient services and some psychological therapies. Psychiatric care remains largely hospital-centered, and long-term hospitalization is common. In recent years, community-based support has gradually expanded, including outreach teams funded by prefectural governments and mental health consultation services at the municipal level^6^. From a policy perspective, Japan’s 8th Medical Care Plan includes provisions for mental health, emphasizing the promotion of community-based care^7^. In addition, the government has issued guidelines for the general public titled “The Mental Health Policy for Workplaces,” which aims to improve mental well-being among workers through preventive measures and support systems^8^.

Despite these institutional frameworks and efforts, significant challenges remain in addressing the burden of schizophrenia and other mental disorders. These include understaffed psychiatric services, inadequate physical healthcare for people with mental illness, slow progress in community transitions, and weak coordination with social services^9^.

This targeted literature review (TLR) aims to comprehensively assess the burdens of schizophrenia in Japan from epidemiological, clinical, societal, economic, and humanistic perspectives. It also seeks to identify stakeholders’ current activities addressing those burdens of disease and unmet needs and to analyze remaining outcome gaps. We hypothesize that despite ongoing efforts, significant gaps remain between stakeholder activities and the multifaceted burdens of schizophrenia.

## Methods

### Data sources

**Fig. 1:**
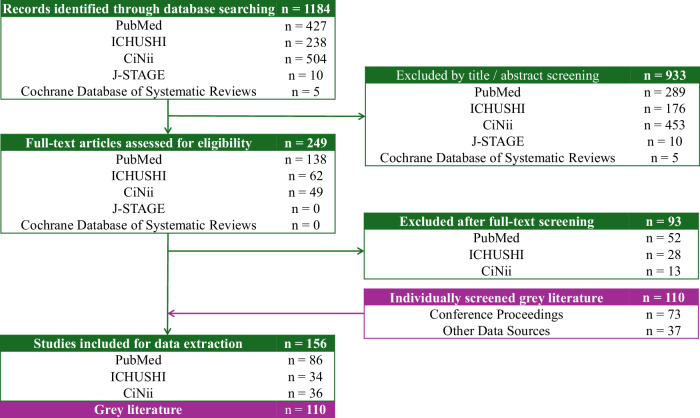
PRISMA flow diagram of study selection process.

This TLR adhered to the Preferred Reporting Items for Systematic Reviews and Meta-Analysis (PRISMA) guidelines^10^, as shown in Fig. 1, and consisted of a biomedical database search and a hand search of gray literature. The database search covering January 1, 2013, to December 31, 2023, was conducted using PubMed, Ichushi, CiNii, J-STAGE, and the Cochrane Database. This 10-year period was chosen to reflect recent trends in research and policy relevant to schizophrenia in Japan.

A hand search of gray literature was conducted to identify relevant evidence in Japan from conference abstracts, government statistics, and organizational reports covering January 1, 2018, to December 31, 2023. Fifteen gray literature sources were selected based on consultation with a clinical expert. The list of sources is presented in Supplementary Table S1. Additionally, stakeholders’ activities were reviewed to clarify their roles and contributions to schizophrenia-related challenges over the past 5 years.

### Inclusion and exclusion criteria

The inclusion and exclusion criteria were defined using the Population, Intervention, Comparators, Outcomes, and Study Design (PICOS) framework as shown in Table 1. This review examined studies on schizophrenia, focusing on patients and caregivers. It included journal and gray literature covering medical treatments, social support, healthcare programs, and policies. The same PICOS criteria were applied to identify stakeholder activities related to schizophrenia.

**Table 1:** Inclusion and exclusion criteria based on the PICOS framework.

Inclusion Criteria	Population: ∙ Patients living with schizophrenia in Japan ∙ Individuals caring for patients with schizophrenia in Japan **I**ntervention & **C**omparators: ∙ Any schizophrenia intervention (medical treatment, social support, healthcare program, healthcare policy) or observation **O**utcome Measures: ∙ Epidemiology: prevalence, incidence, comorbidities, complications, mortality, risk factors ∙ Clinical Management: treatment options, treatment of comorbidities, nursing care, healthcare resource utilization (HCRU), outcomes/prognosis (including relapse prevention, social functions, daily functions, activities of daily living (ADL), social/personal recovery, participation in society) ∙ Society and Welfare: social welfare services, community medicine, peer supporters, stigma, awareness, healthcare policy, multidisciplinary support/team, employment support, housing support, financial support, accessibility to mental health treatment ∙ Humanistic: health-related quality of life (HRQoL), quality-adjusted life year (QALY), patient-reported outcomes (e.g., cognitive impairment, disability, disease burden, recovery) ∙ Economic: economic burden (direct/indirect costs), productivity impact, quality of life impact **S**tudy Type: Journal published studies: ∙ Interventional/empirical studies of healthcare programs ∙ Prospective and retrospective observational studies ∙ Knowledge, Attitude, and Behavior (KAB) studies ∙ Patient/caregiver-centric studies (qualitative studies, surveys, mixed methods, etc.) ∙ Economic evaluation studies, economic models ∙ Systematic reviews, meta-analyses ∙ Treatment guidelines ∙ Other reports (think tanks, policy papers) Gray literature: ∙ Reports ∙ White papers ∙ Conference proceedings ∙ Statements and press releases from patient associations, medical associations, pharma, etc ∙ Published government conference materials
Exclusion Criteria	∙ Studies not involving patients with schizophrenia or their caregivers ∙ Interventions or observations not related to schizophrenia ∙ Studies that do not provide data on epidemiology, clinical management, society and welfare, humanistic, or economic outcomes ∙ Study design other than described in the inclusion criteria

### Data extraction

The data extraction involved reviewing English and Japanese literature from the specified databases and gray literature. The search strategy of databases, with PubMed as a representative example, is outlined in Supplementary Table S2. The review followed PRISMA guidelines, beginning with defining the review’s scope and consensus on search terms. Searches were implemented systematically, and titles and abstracts were screened for relevance. Articles deemed relevant underwent a full-text review, and data were extracted in alignment with the review objectives to ensure the collection of comprehensive and reliable evidence.

For gray literature, conference abstracts were screened like databases, through review of titles and abstracts, followed by data extraction. Other gray literature and stakeholder activities were identified by hand-searching official websites, capturing government, academic, and organizational efforts over the past 5 years to reflect the multifaceted impact of schizophrenia in Japan. These outcomes included epidemiology, clinical management, societal welfare, humanistic outcomes, and economic impact. Each piece of evidence was reviewed and categorized into the most relevant area to facilitate thematic analysis.

## Results

### Identified data

From database searches, 156 articles were included in the analysis. Additionally, 110 gray literature articles were identified through conference proceedings (*n* = 73) and other sources (*n* = 37). The PRISMA flow of the study selection process is shown in Fig. 1. The selected studies are summarized in Tables 2–4. A complete list of all extracted references is provided in Supplementary Document S1. Searches across 15 organizations identified 746 activities related to schizophrenia, as presented in Table 5.

**Table 2:** Summary of published studies stratified by outcome of interest (*n* = 156).

No.^a^	Author, year	Study design	Target population	Study setting	Outcome (category)	Outcome (sub-category)
1	Tsukahara et al. 2023	Retrospective observational	Patient	Hospital(s)	Epidemiology	Risk factor
2	Iwasaki et al. 2023	Qualitative	Caregiver	Hospital(s)	Clinical management	ADL
3	Sato et al. 2023	Quantitative cross-sectional	Patient	Hospital(s)	Clinical management	Health literacy
4	Arai et al. 2023	Retrospective observational	Patient	Hospital(s)	Clinical management	Risk factor
5	Yasui-Furukori et al. 2023	Retrospective observational	Patient	Hospital(s)	Clinical management	Treatment option
6	Onitsuka, 2023	Quantitative cross-sectional	Patient	Hospital(s)	Clinical management	Type of treatment drugs available
7	Tsuboi et al. 2023	Quantitative cross-sectional	Caregiver	Community-dwelling	Humanistic	Burden
8	Hayakawa et al. 2022	Quantitative cross-sectional	Patient	Hospital(s)	Epidemiology	Comorbidities/Complications
9	Watanabe et al. 2022	Quantitative cross-sectional	Patient	Hospital(s)	Epidemiology	Comorbidities/Complications
10	Hagane et al. 2022	Quantitative cross-sectional	Patient	Hospital(s)	Epidemiology	Risk factor
11	Nakagawa et al. 2022	Prospective observational	Patient	Community-dwelling	Clinical management	ADL
12	Kashiwagi et al.^19^	Mixed method	Patient	Database/literature	Clinical management	Hospitalization rate, Duration of Hospitalization
13	Higuchi et al. 2022	Retrospective observational	Patient	Database/literature	Clinical management	Prescribed drugs
14	Okada et al. 2022	Quantitative cross-sectional	Patient	Both of hospital(s) and community-dwelling	Clinical management	Social function
15	Shimada et al. 2022	Quantitative cross-sectional	Patient	Hospital(s)	Clinical management	Social function
16	Uchino et al. 2022	Survey	Patient	Hospital(s)	Society and welfare	Awareness
17	Baba et al.^5^	Quantitative cross-sectional	Patient	ND	Humanistic	HRQoL, WPAI
18	Ishii et al.^48^	Qualitative	Patient	Hospital(s)	Humanistic	QoL
19	Konishi et al. 2021	Retrospective observational	Patient	Database/literature	Epidemiology	In-hospital morbidity.
20	Takahashi et al.^15^	Quantitative cross-sectional	Patient	Database/literature	Epidemiology	Mortality
21	Hori et al. 2021	Retrospective observational	Patient	Hospital(s)	Clinical management	Cognitive impairment
22	Kitamura et al. 2021	Interventional	Both	Hospital(s)	Clinical management	Hospitalization rate
23	Numata et al. 2021	Qualitative	Caregiver	Hospital(s)	Clinical management	Medication behavior
24	Maki et al., 2021	Quantitative cross-sectional	Caregiver	Hospital(s)	Clinical management	Nursing care
25	Usami et al. 2021	Mixed method	Both	Hospital(s)	Clinical management	Nursing care
26	Toyoda et al. 2021	Retrospective observational	Patient	Database/literature	Clinical management	Prescribed drugs
27	Imazu et al. 2021	Retrospective observational	Patient	Database/literature	Clinical management	Side effects/Adverse events
28	Inagaki et al. 2021	SR/Meta-analysis	Patient	Database/literature	Clinical management	Side effects/Adverse events
29	Sato et al. 2021	Retrospective observational	Patient	Both of hospital(s) and community-dwelling	Clinical management	Social function
30	Ichinose et al. 2021	Retrospective observational	Patient	ND	Clinical management	Treatment option
31	Koreki et al. 2021	Quantitative cross-sectional	Patient	Hospital(s)	Clinical management	Type of treatment drugs available
32	Matsuzaki et al. 2021	Retrospective observational	Patient	Hospital(s)	Clinical management	Type of treatment drugs available
33	Arikawa et al.^47^	Retrospective observational	Patient	Hospital(s)	Society and welfare	Financial Support
34	Yasuma et al. 2021	Quantitative cross-sectional	Caregiver	ND	Humanistic	Burden
35	Nagata et al. 2021	Qualitative	Patient	Community-dwelling	Humanistic	PRO
36	Uju et al. 2020	Prospective observational	Patient	Hospital(s)	Epidemiology	Comorbidities/Complications
37	Uju et al. 2020	Quantitative cross-sectional	Patient	Hospital(s)	Epidemiology	Comorbidities/Complications
38	Takahashi et al. 2020	Other reviews/reports	Patient	Database/literature	Epidemiology	Comorbidities/Complications
39	Nishio et al. 2020	Retrospective observational	Patient	Hospital(s)	Epidemiology	Risk factor
40	Inoue et al. 2020	Survey	Patient	Hospital(s)	Epidemiology	Suicide
41	Takahashi et al. 2020	Quantitative cross-sectional	Patient	Hospital(s)	Clinical management	Adherence, other relevant items
42	Hosoi et al.^34^	SR/Meta-analysis	Patient	Database/literature	Clinical management	ADL
43	Nagai et al.^44^	Quantitative cross-sectional	Patient	Hospital(s)	Clinical management	DAI-10 score
44	Ogawa et al. 2020	Survey	Patient	Hospital(s)	Clinical management	Duration of hospitalization
45	Ichimura et al. 2020	Retrospective observational	Patient	Hospital(s)	Clinical management	Other relevant items
46	Hayashi et al. 2020	Interventional	Patient	Hospital(s)	Clinical management	Psychiatric rehabilitation
47	Iwata et al. 2020	Retrospective observational	Patient	Database/literature	Clinical management	Type of treatment drug available
48	Kamei et al. 2020	Survey	Patient	Hospital(s)	Clinical management	Type of treatment drugs available
49	Iwamoto et al. 2020	Qualitative	Caregiver	Hospital(s)	Society and welfare	Peer supporter
50	Yoshida et al. 2020	Qualitative	Patient	Community-dwelling	Society and welfare	Peer supporter
51	Shimada et al. 2020	Retrospective observational	Patient	Both of hospital(s) and community-dwelling	Economic	Hospitalization costs
52	Kasahara-Kiritani, 2020	Economic evaluation study	Patient	ND	Economic	Total costs
53	Toriyama et al. 2019	Retrospective observational	Patient	ND	Epidemiology	Side effects/Adverse events
54	Kaneko et al. 2019	Quantitative cross-sectional	Patient	Hospital(s)	Clinical management	Functional Capacity
55	Nakamura et al. 2019	Economic evaluation study	Patient	ND	Clinical management	Hospitalization rate
56	Doi et al. 2019	Retrospective observational	Patient	Hospital(s)	Clinical management	Type of drugs
57	Sugawara et al.^49^	Quantitative cross-sectional	Patient	Hospital(s)	Clinical management	Type of treatment drugs available
58	Shiosawa et al. 2019	Quantitative cross-sectional	Caregiver	Community-dwelling	Society and welfare	Peer supporter
59	Araki et al. 2019	Survey	Caregiver	Hospital(s)	Society and welfare	Peer supporter
60	Kunizuka et al.^37^	Retrospective observational	Patient	Hospital(s)	Humanistic	Cognitive impairment
61	Watanabe et al. 2019	Survey	Patient	Hospital(s)	Humanistic	PRO
62	Sugawara et al. 2018	SR/Meta-analysis	Patient	Database/literature	Epidemiology	Comorbidities/Complications
63	Hattori et al.^11^	Retrospective observational	Patient	Hospital(s)	Epidemiology	Mortality
64	Tanimoto et al. 2018	Retrospective observational	Patient	Hospital(s)	Epidemiology	Suicide attempt
65	Ebisu et al. 2018	Retrospective observational	Patient	Hospital(s)	Clinical management	Duration of hospitalization
66	Matsumoto et al. 2018	Qualitative	Caregiver	Hospital(s)	Clinical management	Nursing care
67	Haga et al.^17^	Retrospective observational	Patient	Hospital(s)	Clinical management	Treatment option, risk factor
68	Inada et al. 2018	Retrospective observational	Patient	Hospital(s)	Clinical management	Type of treatment drugs available
69	Maki et al. 2018	Qualitative	Caregiver	Hospital(s)	Society and welfare	Peer supporter
70	Matsumoto et al. 2018	Qualitative	Caregiver	Community-dwelling	Society and welfare	Support
71	Fujino et al. 2018	Survey	Patient	Hospital(s)	Humanistic	Cognitive impairment
72	Kageyama et al., 2018	Survey	Caregiver	Community-dwelling	Humanistic	CRO
73	Kageyama et al, 2018	Survey	Caregiver	Community-dwelling	Humanistic	PRO
74	Kasuga et al. 2018	Qualitative	Patient	Community-dwelling	Humanistic	PRO
75	Sugawara et al. 2018	Qualitative	Patient	Hospital(s)	Humanistic	PRO
76	Kamiura et al.^60^	Economic evaluation study	Patient	ND	Economic	ICER
77	Sruamsiri et al., 2018	Economic evaluation study	Caregiver	Database/literature	Economic	Productivity
78	Omi et al. 2017	Retrospective observational	Patient	Hospital(s)	Clinical management	Duration of hospitalization
79	Uchida et al. 2017	Quantitative cross-sectional	Patient	Hospital(s)	Clinical management	Duration of hospitalization
80	Cheung et al.^18^	Retrospective observational	Patient	Database/literature	Clinical management	Hospitalization rate, ER visit
81	Hatano et al. 2017	Survey	Patient	Hospital(s)	Clinical management	Side effects/Adverse events
82	Miyauchi et al. 2017	Retrospective observational	Patient	Hospital(s)	Clinical management	Type of treatment drugs available
83	Narita et al. 2017	Mixed method	Patient	Community-dwelling	Society and welfare	Community medicine
84	Niimura et al.^46^	Quantitative cross-sectional	Patient	Both of hospital(s) and community-dwelling	Society and welfare	Social welfare service
85	Fujino et al.^36^	Survey	Patient	Hospital(s)	Humanistic	Cognitive impairment
86	Kiriyama et al. 2017	Mixed method	Both	Hospital(s)	Humanistic	PRO
87	Setoguchi et al. 2017	SR/Meta-analysis	Patient	Database/literature	Humanistic	PRO
88	Fujimoto et al. 2017	Survey	Patient	Community-dwelling	Humanistic	PRO
89	Nakamura et al.^61^	Economic evaluation study	Patient	Hospital(s)	Economic	Total costs
90	Sado et al.^31^	Quantitative cross-sectional	Patient	Hospital(s)	Epidemiology	Comorbidities/Complications
91	Sugai et al.^16^	Quantitative cross-sectional	Patient	Hospital(s)	Epidemiology	Comorbidities/Complications
92	Sugai et al.^16^	Quantitative cross-sectional	Patient	Hospital(s)	Epidemiology	Comorbidities/Complications
93	Ishikawa et al.^13^	Retrospective observational	Patient	Hospital(s)	Epidemiology	Mortality
94	Sugawara et al. 2016	Quantitative cross-sectional	Patient	Hospital(s)	Epidemiology	Risk factor
95	Yoshida et al.^51^	Quantitative cross-sectional	Patient	Both of hospital(s) and community-dwelling	Epidemiology	Risk factor
96	Tarutani et al. 2016	Survey	Patient	Hospital(s)	Clinical management	Adherence
97	Shimada et al. 2016	Retrospective observational	Patient	Hospital(s)	Clinical management	Hospitalization rate
98	Takeuchi et al, 2016	Quantitative cross-sectional	Both	Hospital(s)	Clinical management	Other relevant items
99	Akiyama et al. 2016	Other reviews/reports	Patient	Hospital(s)	Clinical management	Social function
100	Hashimoto et al. 2016	Quantitative cross-sectional	Patient	Hospital(s)	Clinical management	Type of treatment drugs available
101	Kageyama et al., 2016	Survey	Caregiver	Community-dwelling	Humanistic	CRO
102	Niimura et al.^53^	Qualitative	Patient	Hospital(s)	Humanistic	PRO
103	Iinuma et al. 2016	Mixed method	Patient	Hospital(s)	Humanistic	PRO
104	Kawanabe et al. 2016	Survey	Patient	Hospital(s)	Humanistic	QoL
105	Hashimoto et al. 2016	Other reviews/reports	Patient	Hospital(s)	Economic	Medicine costs
106	Ito et al. 2015	Quantitative cross-sectional	Patient	Both of hospital(s) and community-dwelling	Epidemiology	Comorbidities/Complications
107	Kanzaki et al. 2015	Quantitative cross-sectional	Patient	Hospital(s)	Epidemiology	Comorbidities/Complications
108	Sugai et al. 2015	Quantitative cross-sectional	Patient	Hospital(s)	Epidemiology	Comorbidities/Complications
109	Nakamura et al. 2015	Survey	Patient	Hospital(s)	Epidemiology	Comorbidities/Complications
110	Ikeda et al. 2020	Prospective observational	Patient	Hospital(s)	Clinical management	Duration of hospitalization
111	Tachimori et al. 2015	Retrospective observational	Patient	Hospital(s)	Clinical management	Duration of hospitalization
112	Kuwabara et al.^21^	Retrospective observational	Patient	Database/literature	Clinical management	Hospitalization rate
113	Takahashi et al. 2015	Prospective observational	Patient	Hospital(s)	Clinical management	Hospitalization rate (remission)
114	Sumiyoshi et al. 2015	Quantitative cross-sectional	Patient	Hospital(s)	Clinical management	Social function
115	Sasamoto et al. 2015	Qualitative	Caregiver	Hospital(s)	Society and welfare	Multidisciplinary support
116	Yamada^42^	Survey	Patient	Hospital(s)	Society and welfare	Stigma
117	Sasaki et al. 2015	Mixed method	Patient	Hospital(s)	Humanistic	PRO
118	Nagasawa et al. 2015	Mixed method	Patient	Hospital(s)	Humanistic	PRO
119	Kimbara et al.^12^	Retrospective observational	Patient	Hospital(s)	Epidemiology	Comorbidities/Complications
120	Imai et al. 2014	Other reviews/reports	Patient	Hospital(s)	Epidemiology	Comorbidities/Complications
121	Sugawara et al. 2014	Quantitative cross-sectional	Patient	Hospital(s)	Epidemiology	Comorbidities/Complications
122	Suzuki et al. 2014	Quantitative cross-sectional	Patient	Hospital(s)	Epidemiology	Comorbidities/Complications
123	Harada et al. 2014	Other reviews/reports	Patient	Hospital(s)	Epidemiology	Suicide attempt
124	Ikeshita et al. 2014	Retrospective observational	Patient	Hospital(s)	Epidemiology	Suicide attempt
125	Ishii et al. 2014	Other reviews/reports	Patient	Hospital(s)	Epidemiology	Suicide attempt
126	Teraishi et al, 2014	Survey	Patient	Hospital(s)	Epidemiology	Suicide attempt
127	Sugibayashi et al.^20^	Quantitative cross-sectional	Patient	Hospital(s)	Clinical management	ADL
128	Arai^14^	Survey	Both	Hospital(s)	Clinical management	Nursing care
129	Ochiai et al. 2014	Retrospective observational	Patient	Database/literature	Clinical management	Prescribed drugs
130	Omi et al. 2014	Survey	Patient	Hospital(s)	Clinical management	Social function
131	Inoue et al. 2014	Retrospective observational	Patient	Hospital(s)	Clinical management	Social function
132	Tamura et al.^54^	Qualitative	Patient	Hospital(s)	Humanistic	PRO
133	Nakamura et al. 2014	Quantitative cross-sectional	Patient	Community-dwelling	Humanistic	QoL
134	Inamura et al. 2013	Quantitative cross-sectional	Patient	Hospital(s)	Epidemiology	Comorbidities/Complications
135	Suzuki et al. 2013	Retrospective observational	Patient	Hospital(s)	Epidemiology	Comorbidities/Complications
136	Suzuki et al. 2013	Quantitative cross-sectional	Patient	Hospital(s)	Epidemiology	Comorbidities/Complications
137	Umene-Nakano et al. 2013	Quantitative cross-sectional	Patient	Hospital(s)	Epidemiology	Comorbidities/Complications
138	Inamura, 2013	Quantitative cross-sectional	Patient	Hospital(s)	Epidemiology	Comorbidities/Complications
139	Sugawara et al.^50^	Quantitative cross-sectional	Patient	Hospital(s)	Epidemiology	Comorbidities/Complications, HRQoL
140	Saito et al.^32^	Retrospective observational	Patient	Hospital(s)	Epidemiology	Risk factor
141	Kimura et al. 2013	Retrospective observational	Patient	Hospital(s)	Epidemiology	Suicide attempt
142	Saito et al.^32^	Retrospective observational	Patient	Hospital(s)	Clinical management	ADL
143	Tanioka et al. 2013	Retrospective observational	Patient	Hospital(s)	Clinical management	Duration of hospitalization
144	Sato et al. 2013	Qualitative	Patient	Hospital(s)	Clinical management	Duration of hospitalization
145	Tamasato, 2013	Qualitative	Patient	Hospital(s)	Clinical management	Nursing care
146	Onose et al. 2013	Qualitative	Patient	Hospital(s)	Clinical management	Social and personal recovery
147	Takahashi et al. 2013	Prospective observational	Patient	Hospital(s)	Clinical management	Type of treatment drugs available
148	Okumura et al. 2013	Retrospective observational	Patient	Database/literature	Clinical management	Type of treatment drugs available
149	Kurosawa et al. 2013	Retrospective observational	Patient	Database/literature	Clinical management	Type of treatment drugs available
150	Yoshimura, 2013	Qualitative	Caregiver	Hospital(s)	Society and welfare	Peer supporter
151	Iwasaki et al. 2013	Qualitative	Caregiver	Community-dwelling	Society and welfare	Peer supporter
152	Kagawa et al. 2013	Qualitative	Caregiver	Hospital(s)	Society and welfare	Peer supporter
153	Fujita et al. 2013	Interventional	Patient	Hospital(s)	Humanistic	Health state utitlities
154	Koyama, 2013	Mixed method	Patient	Community-dwelling	Humanistic	PRO
155	Kikuchi, 2013	Qualitative	Patient	Community-dwelling	Humanistic	PRO
156	Sado et al.^59^	Economic evaluation study	Patient	ND	Economic	Total costs

*NA* not applicable, *ND* no data, *ADL* activities of daily living, *CRO* caregiver-reported outcome, *DAI-10* drug attitude inventory-10, *ER* emergency room, *HRQoL* health-related quality of life, *ICER* incremental cost-effectiveness ratio, *PRO* patient-reported outcome, *QoL* quality of life, *WPAI* Work Productivity and Activity Impairment.

^a^Note: All literature sources referenced in this table can be found in Supplementary Document S1.

**Table 3:** Summary of conference abstracts stratified by outcome of interest (*n* = 73).

No.^a^	Author, year	Study design	Target population	Study setting	Outcome (category)	Outcome (sub-category)
157	Kamei et al. 2023	Retrospective observational	Patient	Hospital(s)	Epidemiology	Comorbidities/Complications
158	Goto et al.^27^	Qualitative	Patient	Community-dwelling	Clinical management	Burden of treatment
159	Ida et al. 2023	Retrospective observational	Patient	Hospital(s)	Clinical management	Management of comorbidities/complications
160	Mochizuki et al. 2023	Qualitative	Patient	Community-dwelling	Clinical management	Nursing care
161	Narita et al. 2023	Retrospective observational	Patient	Hospital(s)	Clinical management	Prescription
162	Matsuda et al. 2023	Retrospective observational	Patient	Hospital(s)	Clinical management	Prescription
163	Takagi et al. 2023	Quantitative cross-sectional	Caregiver	Community-dwelling	Humanistic	CRO
164	Watabe, 2023	Quantitative cross-sectional	Caregiver	Community-dwelling	Humanistic	CRO
165	Nakai et al. 2023	Qualitative	Patient	Community-dwelling	Humanistic	PRO
166	Tsutsumi et al. 2022	Retrospective observational	Patient	Hospital(s)	Epidemiology	Comorbidities/Complications
167	Fukushima et al. 2022	Survey	Patient	ND	Clinical management	HCRU
168	Nakamura et al. 2022	Survey	Patient	ND	Clinical management	Prescription
169	Shimada et al. 2022	Quantitative cross-sectional	Patient	Hospital(s)	Clinical management	Social function
170	Takemura et al. 2022	Other reviews/reports	Patient	Hospital(s)	Clinical management	Treatment option
171	Kobatake et al. 2022	Other reviews/reports	Patient	Database/literature	Society and welfare	Self-stigma
172	Hiyama et al. 2022	Qualitative	Caregiver	Community-dwelling	Society and welfare	Support
173	Nishi et al. 2022	Quantitative cross-sectional	Patient	Community-dwelling	Humanistic	PRO
174	Fukuda et al. 2022	Quantitative cross-sectional	Patient	Community-dwelling	Humanistic	PRO
175	Suzuki et al. 2022	Quantitative cross-sectional	Patient	Hospital(s)	Humanistic	PRO
176	Watanabe et al. 2022	Quantitative cross-sectional	Patient	Hospital(s)	Humanistic	PRO
177	Yokota et al. 2021	Retrospective observational	Patient	Hospital(s)	Epidemiology	Comorbidities/Complications
178	Hirota et al. 2021	Retrospective observational	Patient	ND	Clinical management	Burden of treatment
179	Taniguchi et al.^29^	Retrospective observational	Patient	ND	Clinical management	Burden of treatment
180	Kida et al.^22^	Retrospective observational	Patient	Hospital(s)	Clinical management	Management of comorbidities/complications
181	Ono et al. 2021	Retrospective observational	Patient	Hospital(s)	Clinical management	Maternal-fetal management
182	Suzuki et al. 2021	Qualitative	Caregiver	Hospital(s)	Clinical management	Nursing care
183	Arai et al. 2021	Qualitative	Caregiver	Hospital(s)	Clinical management	Nursing care
184	Kimura^56^	Other reviews/reports	Caregiver	Database/literature	Clinical management	Nursing care
185	Nishiyama et al. 2021	Retrospective observational	Patient	Hospital(s)	Clinical management	Prescription
186	Furuhata et al.^26^	Survey	Patient	Hospital(s)	Clinical management	Prescription
187	Moriwaki et al.^39^	Retrospective observational	Patient	ND	Clinical management	Relationship between COVID-19 and mental illness
188	Hanawa et al.^40^	Retrospective observational	Patient	ND	Clinical management	Relationship between COVID-19 and mental illness
189	Inagawa et al. 2021	Interventional	Patient	ND	Clinical management	Treatment option
190	Funai et al. 2021	Interventional	Patient	Hospital(s)	Clinical management	Treatment option
191	Inuyama et al. 2021	Retrospective observational	Patient	ND	Clinical management	Treatment option
192	Kojima et al.^55^	Qualitative	Caregiver	Community-dwelling	Humanistic	CRO
193	Kimura et al.^56^	Qualitative	Caregiver	ND	Humanistic	CRO
194	Koyama et al. 2021	Quantitative cross-sectional	Patient	Hospital(s)	Humanistic	PRO
195	Ogata et al. 2021	Quantitative cross-sectional	Patient	Both of hospital(s) and community-dwelling	Humanistic	PRO
196	Yamaguchi et al. 2021	Quantitative cross-sectional	Patient	Hospital(s)	Humanistic	PRO
197	Ishii, 2020	Prospective observational	Patient	Hospital(s)	Clinical management	Burden of treatment
198	Sugai et al.^23^	Prospective observational	Patient	Hospital(s)	Clinical management	Management of comorbidities/complications
199	Inakuma et al.^24^	Retrospective observational	Patient	Hospital(s)	Clinical management	Management of comorbidities/complications
200	Akasaka et al. 2020	Quantitative cross-sectional	Patient	ND	Clinical management	Management of comorbidities/complications
201	Kobayashi et al. 2020	Retrospective observational	Patient	Hospital(s)	Clinical management	Maternal-fetal management
202	Aoki et al. 2020	Retrospective observational	Patient	ND	Clinical management	Treatment option
203	Tanaka, 2020	Retrospective observational	Patient	Hospital(s)	Clinical management	Treatment option
204	Kitamura et al. 2019	Survey	Patient	Hospital(s)	Epidemiology	Comorbidities/Complications
205	Matsui et al. 2019	Retrospective observational	Patient	Database/literature	Epidemiology	Comorbidities/Complications
206	Onda et al. 2019	Survey	Patient	Hospital(s)	Epidemiology	Comorbidities/Complications
207	Sugai et al. 2019	Quantitative cross-sectional	Patient	Hospital(s)	Epidemiology	Comorbidities/Complications
208	Takahashi et al. 2019	Survey	Patient	ND	Clinical management	HCRU
209	Masaki et al. 2019	Survey	Patient	Hospital(s)	Clinical management	HCRU
210	Fukuda et al. 2019	Quantitative cross-sectional	Patient	Hospital(s)	Clinical management	HCRU
211	Suzuki et al. 2019	Retrospective observational	Patient	Hospital(s)	Clinical management	Lifestyle management
212	Fujiwara et al. 2019	Prospective observational	Patient	Hospital(s)	Clinical management	Lifestyle management
213	Kusumi et al.^25^	Prospective observational	Patient	Database/literature	Clinical management	Management of comorbidities/complications
214	Ono et al. 2019	Quantitative cross-sectional	Patient	Hospital(s)	Clinical management	Management of comorbidities/complications
215	Otake et al. 2019	Quantitative cross-sectional	Patient	Hospital(s)	Clinical management	Management of comorbidities/complications
216	Arai et al. 2019	Qualitative	Caregiver	Hospital(s)	Clinical management	Nursing care
217	Ishii, 2019	Qualitative	Caregiver	Hospital(s)	Clinical management	Nursing care
218	Kikuchi et al. 2019	Retrospective observational	Patient	Hospital(s)	Clinical management	Prescription
219	Ozeki et al. 2019	Retrospective observational	Patient	ND	Clinical management	Prevention of relapse
220	Watabe, 2019	Interventional	Caregiver	Hospital(s)	Clinical management	Treatment option
221	Naganuma et al. 2019	Other reviews/reports	Patient	Hospital(s)	Clinical management	Treatment option
222	Sakayori et al. 2019	Survey	Patient	Hospital(s)	Clinical management	Treatment option
223	Kusuno, 2019	Other reviews/reports	Patient	Community-dwelling	Society and welfare	Accessibility to treatment of mental health
224	Nemoto et al. 2019	Prospective observational	Patient	ND	Society and welfare	Social function
225	Imaeda et al. 2019	Qualitative	Patient	Both of hospital(s) and community-dwelling	Society and welfare	Support
226	Yabuta^57^	Qualitative	Caregiver	Community-dwelling	Humanistic	CRO
227	Fujisawa^58^	Qualitative	Caregiver	Community-dwelling	Humanistic	CRO
228	Moriguchi et al. 2019	Quantitative cross-sectional	Patient	Community-dwelling	Humanistic	PRO
229	Nakamura et al. 2019	Quantitative cross-sectional	Patient	Community-dwelling	Humanistic	PRO

*NA* not applicable, *ND* no data, *COVID-19* Coronavirus Disease 2019, *CRO* caregiver-reported outcome, *HCRU* healthcare resource utilization, *PRO* patient-reported outcome.

^a^Note: All literature sources referenced in this table can be found in Supplementary Document S1.

**Table 4:** Summary of other source stratified by outcome of interest (*n* = 37).

No.^a^	Author/Publisher, year	Type of literature	Data source	Target population	Outcome (category)	Outcome (sub-category)
230	MHLW, 2023	Government statistics	MHLW	Patient	Clinical management	Number of hospitalized patients
231	NFAFMJ, 2023	Report	NFAFMJ	Caregiver	Humanistic	CRO
232	MHLW, 2023	Government statistics	MHLW	Patient	Economic	Medical fee
233	MHLW, 2022	Government statistics	MHLW	Patient	Epidemiology	Patient population
234	MHLW, 2022	Government statistics	MHLW	Patient	Epidemiology	Patient population
235	MHLW, 2022	Government statistics	MHLW	Patient	Epidemiology	Patient population
236	MHLW, 2022	Government statistics	MHLW	Patient	Epidemiology	Patient population
237	MHLW, 2022	Government statistics	MHLW	Patient	Clinical management	Medical fee
238	MHLW, 2022	Government statistics	MHLW	Patient	Clinical management	Number of hospitalized patients
239	MHLW, 2023	Government statistics	MHLW	Patient	Clinical management	Nursing care
240	MHLW, 2022	Government statistics	MHLW	Patient	Clinical management	Treatment rate
241	MHLW, 2022	Government statistics	MHLW	Patient	Clinical management	Treatment rate
242	MHLW, 2022	Government statistics	MHLW	Patient	Economic	Medical fee
243	MHLW, 2021	Government statistics	MHLW	Patient	Clinical management	HCRU
244	MHLW, 2021	Government statistics	MHLW	Patient	Clinical management	HCRU
245	MHLW, 2021	Government statistics	MHLW	Patient	Clinical management	HCRU
246	MHLW, 2021	Government statistics	MHLW	Patient	Clinical management	HCRU
247	MHLW, 2021	Government statistics	MHLW	Patient	Clinical management	HCRU
248	MHLW, 2021	Government statistics	MHLW	Patient	Clinical management	Medical fee
249	MHLW, 2021	Government statistics	MHLW	Patient	Clinical management	Number of hospitalized patients
250	MHLW, 2021	Government statistics	MHLW	Patient	Economic	Medical expenses
251	MHLW, 2021	Government statistics	MHLW	Patient	Economic	Medical fee
252	MHLW, 2020	Government statistics	MHLW	Patient	Clinical management	HCRU
253	MHLW, 2020	Government statistics	MHLW	Patient	Clinical management	HCRU
254	MHLW, 2020	Government statistics	MHLW	Patient	Clinical management	HCRU
255	MHLW, 2020	Government statistics	MHLW	Patient	Clinical management	HCRU
256	MHLW, 2020	Government statistics	MHLW	Patient	Clinical management	HCRU
257	MHLW, 2020	Government statistics	MHLW	Patient	Clinical management	HCRU
258	MHLW, 2020	Government statistics	MHLW	Patient	Clinical management	Medical fee
259	MHLW, 2020	Government statistics	MHLW	Patient	Clinical management	Number of hospitalized patients
260	MHLW, 2020	Government statistics	MHLW	Patient	Clinical management	Nursing care
261	MHLW, 2020	Government statistics	MHLW	Patient	Economic	Medical expenses
262	MHLW, 2020	Government statistics	MHLW	Patient	Economic	Medical fee
263	MHLW, 2019	Government statistics	MHLW	Patient	Clinical management	Medical fee
264	MHLW, 2019	Government statistics	MHLW	Patient	Economic	Medical expenses
265	MHLW, 2018	Government statistics	MHLW	Patient	Clinical management	Medical fee
266	MHLW, 2018	Government statistics	MHLW	Patient	Economic	Medical expenses

*CRO* caregiver-reported outcome, *HCRU* healthcare resource utilization, *MHLW* Ministry of Health, Labor, and Welfare, *NFAFMJ* The National Federation of Associations of Families with the Mental Illness in Japan.

^a^Note: All literature sources referenced in this table can be found in Supplementary Document S1.

**Table 5:** Overview of stakeholder activities.

Stakeholder	Type of activities, hit numbers (%)						
	Academic/Research	Education/Training	Publication	Information/Communication	Policy/Advocacy	Community support and services	Total
Japanese Society of Schizophrenia Research	0	0	1	18	0	0	19
Japanese Society of Psychiatry and Neurology	0	0	0	0	0	0	0
Japan Academy of Psychiatric and Mental Health Nursing	6	0	0	7	0	0	13
Japanese Society for Social Psychiatry	0	0	2	0	0	0	2
Japanese Society of Neuropsychopharmacology	0	1	3	11	0	0	15
Japanese Association for Emergency Psychiatry	3	0	1	0	0	0	4
National Center of Neurology and Psychiatry	29	4	0	25	0	0	58
Evidence-based Information site on community lives for people with mental illness	0	4	0	22	0	0	26
Health and Global Policy Institute	0	0	0	1	1	0	2
Ministry of Health, Labor, and Welfare (MHLW)	11	0	0	7	132	0	150
The National Federation of Associations of Families with Mental Illness in Japan	1	0	0	8	2	0	11
Porque (Organization of Persons with Psychosocial Disabilities)	0	1	4	35	2	0	42
COMHBO (Community Mental Health and Welfare Organization)	0	0	0	10	0	0	10
Smile Navigator	0	0	0	14	0	0	14
Kokoro-share	0	0	0	7	0	0	7

Note: Academic/Research: overall academic and research activities, including research, conferences, presentations, and other activities promoting collaboration between organizations. Education/Training: overall activities related to education and training, including school education. Publication: overall activities of publishing printed materials, like association journals. Information/Communication: overall activities related to providing information, including outreaching, hosting lectures, and organizing symposiums. Policy/Advocacy: overall activities related to policy, including advocacy and activities targeting specific policies. Community support and services: overall activities related to support, such as building networks, disability welfare services, community life support, counseling support, and work support.

### Characteristics of included studies

Most studies focused on patients (*n* = 132), followed by caregivers (*n* = 19) and both (*n* = 5). Clinical burden was most common (*n* = 66), followed by epidemiology (*n* = 41), humanistic burden (*n* = 27), societal and welfare burden (*n* = 15), and economic burden (*n* = 7). Publications were from 2013 to 2017 (*n* = 79) and 2018 to 2023 (*n* = 77). Since the peak in 2013 (*n* = 23), the number of publications has shown a declining trend. In gray literature, conference abstracts mainly targeted patients (*n* = 60), focusing on clinical management (*n* = 46), humanistic burden (*n* = 16), epidemiology (*n* = 7), and societal and welfare burden (*n* = 4). Publication years ranged from 2019 to 2023. Other sources also targeted patients (*n* = 35), addressing clinical management (*n* = 24), economic burden (*n* = 8), epidemiology (*n* = 4), and humanistic burden (*n* = 1).

### Epidemiology

A self-reported online survey from the National Health and Wellness Survey reported a prevalence of 0.59%, primarily among individuals aged 18–64 (89.3%)^5^. Five studies reported data on mortality^11–15^. A single-center study by Hattori et al. followed up 59 patients (mean age 64.9) for 10 years; 11 died (mean age 70.6)^11^. Kimbara et al. examined 134 inpatients (mean age 63); 12 died over 5 years, nearly half (*n* = 7) from pneumonia^12^. Ishikawa et al. used data from the Japanese Diagnosis Procedure Combination (DPC) database to assess schizophrenia and cancer stages in acute hospitals; the 30-day in-hospital mortality rate of cancer patients with schizophrenia was 4.2%^13^. Arai’s dual-center study reported that all 16 patients with schizophrenia and cancer died within a year of the diagnosis; diagnoses were at advanced stages^14^. Takahashi et al. reported in-hospital mortality rates of 12.9% (acute myocardial infarction: AMI) and 5.8% (pulmonary embolism) among schizophrenia patients using DPC data^15^.

Comorbidities or complications, such as obesity, hypertension, depression, congestive heart failure, and type 2 diabetes mellitus, were consistently highlighted. A multi-center study found hypertension in 30.5% of outpatients and 19.9% of inpatients; diabetes in 16.8% and 7.1%, respectively^16^. A single-center study by Haga et al. identified pneumonia risk factors: age, underweight status, smoking habit, use of atypical antipsychotics, and high doses^17^. A database study by Cheung et al. on medical information from acute care hospitals in Japan reported that 17.2% of 45,201 schizophrenia patients were also diagnosed with depression^18^.

### Clinical management burden

The database study by Cheung et al. (study period between July 2013 and June 2015) reported a mean of 2.0 hospitalizations among 45,201 schizophrenia patients^18^. Similarly, Kashiwagi et al. analyzed data from the Cognitive Genetics Collaborative Research Organization database (study period: October 2005 and October 2019) and found patients with a violence history had a mean of 2.3 hospitalizations vs. 1.2 without violence^19^. Furthermore, hospitalization duration varied widely, from 20.7 days to 13,833.5 days. Sugibayashi et al. reported a mean of 3242.1 days among 8379 inpatients (4452 males, 2691 over 65 years old)^20^. A database study by Kuwabara analyzed 657 patients (222 males) from MDV; the mean index hospitalization was 84.3 days^21^.

Studies on comorbidities and complications often explored links between antipsychotic drug prescription and comorbidities and complications^22–25^. Sugai et al. found that increased prescriptions worsened body mass index, systolic blood pressure, low-density lipoprotein cholesterol, and fasting blood sugar values. They also examined prescribing practices and medication adherence^23^. Furuhata et al. found multiple sleep medications were associated with age, polypharmacy, and mood stabilizers^26^. Other studies involved medication adherence, challenges of isolation and physical restraint in psychiatric wards, and challenges in physical comorbidity wards^27–30^.

Six studies reported activities of daily living (ADL)^20,31–35^. A single-center study found that among 770 patients, 513 had mild, 137 moderate, and 81 severe ADL disorders^31^. In another single-center study, Saito et al. found that 17.6% of 272 schizophrenia patients with dysphagia were “independent” in daily activities^32^.

Three studies examined cognitive impairment^36–38^. A multi-center study by Fujino et al. reported that the average cognitive impairment score of patients was −16.3, lower than the pivotal score of −10, significantly lower than healthy controls. The score was calculated as the difference between the current Full-Scale Intelligence Quotient (IQ) assessed by the Wechsler Adult Intelligence Scale - Second Edition and the premorbid IQ estimated using the Japanese Adult Reading Test - 25^36^. A single-center study by Kunizuka et al. found a composite *z*-score of −2.1 using the Brief Assessment of Cognition in Schizophrenia-Japanese version^37^. Hori et al. found composite *z*-scores of −0.7, −1.1, −1.5, and −1.8 among patients with 0, 1, 2, and 3 hospitalizations, respectively^38^.

According to three gray literature articles, the COVID-19 pandemic had notable impacts on schizophrenia patients. Moriwaki et al. reported increased self-harm and suicidal ideation among hospitalized patients in 2020, although treatment outcomes remained stable and hospitalization duration shortened^39^. Hanawa et al. identified schizophrenia among suicide attempt diagnoses related to COVID-19-related anxiety and economic stress^40^. Fukushima et al. reported that in an acute psychiatric ward, a 23% increase in discharged schizophrenia patients in 2020 (from 92 to 113), while the average stay decreased by about 10 days to 58 days^41^.

### Society and welfare burden

Yamada assessed self-stigma using the Perceived Devaluation Discrimination (PDD) scale developed a self-reported scale with 12 items to evaluate perceived stigma, discrimination, stereotyping, and social rejection^42,43^. The scale ranges from 12 to 60, with higher scores indicating lower levels of stigma. Among 97 inpatients, the mean PDD score was 30.5, lower than those reported in two other studies (mean scores: 32.6 and 31.5). Nagai et al. conducted a cross-sectional study on VAGUS to assess schizophrenia awareness among 148 outpatients, reporting a mean score of 6.6 (standard deviation of 1.5)^44^. VAGUS is a standardized tool to assess how emotional conditions might influence behavior and decision-making, consisting of 10 self-report and five clinician-rated questions^45^. A cross-sectional study by Niimura et al. using a patient survey reported a financial burden. Among 21,101 patients with less than 80 days of hospitalization, 19.4% received public assistance. Similarly, among 21,167 patients with over 80 days of hospitalization, 19.1% used public assistance^46^. A single-center study by Arikawa et al. reported that 151 out of 437 schizophrenia patients used medical expenses for services and support for disabilities. The study mentioned that financial support improves persistence of outpatient treatment for patients with schizophrenia^47^.

### Humanistic burden

Among six studies^5,48–52^, four quality of life (QoL) assessment tools were identified: EuroQol 5-Dimension (EQ-5D), the Japanese version of the Schizophrenia Quality of Life Scale (JSQLS), MOS 36-Item Short-Form Health Survey (SF-36) v2, and SF-12v2. Baba et al. evaluated Health-related Quality of Life (HRQoL) of schizophrenia patients using SF-12v2, EQ-5D Index, and EQ-5D VAS, dividing patients based on their Patient Health Questionnaire-9 (PHQ-9) scores (<10 vs. ≥10). Patients with PHQ-9 < 10 showed high HRQoL, with EQ-5D VAS scores of 67.8, while those with PHQ-9 ≥ 10 had moderate HRQoL with EQ-5D VAS scores of 44.3. SF-12v2 scores indicated slightly below average physical functioning for both groups, with better mental health for those with PHQ-9 scores < 10^5^. Another study by Ishii et al. with 50 patients used JSQLS to evaluate QoL, with a mean of 45.0 for psychosocial, 47.3 for motivation and energy, and 34.9 for symptoms and side effects scales^48^. The study also reported the finding that depressive symptoms are key predictors of subjective QoL, highlighting the importance of addressing depression to improve overall well-being in schizophrenia patients.

A qualitative study by Niimura et al. involving 18 discharged patients identified challenges such as “separating life as an inpatient from community life,” including dissatisfaction with inpatient care and a lack of ability to coordinate post-discharge life. Patients reported dissatisfaction with the admission process and treatment in protection rooms, and challenges in managing life after discharge^53^. Another qualitative study by Tamura et al. with three patients assessed the impact of clozapine treatment, identifying themes like anxiety about a new medication, the hardship of frequent outpatient visits, and joy of symptom attenuation post-treatment^54^. Qualitative studies also highlighted difficulties faced by caregivers, particularly parents and siblings, in supporting patients. Parents struggled to accept their child’s illness and were anxious about their child’s job continuity despite regular employment^55–58^.

### Economic burden

An economic evaluation study estimated the cost of schizophrenia in 2008 from a societal perspective. Direct, indirect, and total costs were Japanese yen (JPY) 770.0 billion (US dollars [USD] 5.1 billion), JPY 2.0 trillion (USD 13.2 billion), and JPY 2.8 trillion (USD 18.3 billion), respectively^59^. Direct costs included social services and healthcare under health insurance, involuntary admission, medical care, and supervision act costs. Indirect costs included morbidity costs (e.g., loss of income due to hospitalization and hospital visits) and mortality costs (future income lost due to early death due to a certain illness). Inpatient and outpatient costs were JPY 602.8 billion (USD 4.0 billion) and 148.0 billion (USD 1.0 billion), respectively^59^. Kamiura et al. estimated the cost-effectiveness of oral medication by hazard ratios for hospitalization compared to LAI. They found the largest reduction of incremental cost-effectiveness ratios between hazard ratios of 1.1 and 2.0^60^. Baba et al. assessed the productivity loss of schizophrenia patients using the Work Productivity and Activity Impairment (WPAI) tool. The mean total activity impairment was 22.8 for PHQ-9 scores <10 and 53.6 for scores ≥10, indicating moderate to significant impairment^5^. Nakamura et al. estimated productivity losses due to unemployment or premature death, totaling JPY 3990 million (USD 26.3 million). 4146 patients’ labor was lost due to unemployment, and 151 patients due to premature death^61^. For caregivers, Sruamsiri et al. analyzed productivity losses among caregivers. Using the WPAI questionnaire with 171 caregivers, they found absenteeism at 4.7% and presenteeism at 25.0%, resulting in per capita annual productivity costs of JPY 2.4 million (USD 16.0 thousand), with presenteeism accounting for 97% (JPY 2.4 million [USD 15.6 thousand]). They also calculated annual productivity losses due to job resignation for caregiving at JPY 1.3 million (USD 8.6 thousand), leading to a total annual productivity loss of JPY 2.4 million (USD 15.8 thousand)^62^.

The economic outcomes reported in Japanese yen are accompanied by their equivalent values in USD in parentheses, calculated using the exchange rate as of February 28, 2025, which represents the conversion rate at the time of writing this manuscript. The rate applied for conversion was 1 JPY = 0.0066 USD.

### Stakeholder activities

Stakeholder activities were categorized into six main types: academic and research activities, education and training, publication, information and communication, policy and advocacy, and community support and services. The Ministry of Health, Labour, and Welfare (MHLW) was the most active organization, with 150 activities, predominantly in policy and advocacy. Efforts also included research on clozapine and medical fees. The National Center of Neurology and Psychiatry was the second-most active organization, with 58 activities focused on academic research, information dissemination, and other areas. Also, they led educational efforts for psychiatrists through the Effectiveness of GUIdeline for Dissemination and Education in psychiatric treatment (EGUIDE) project. The Japanese Society of Neuropsychopharmacology primarily disseminated information and updated treatment guidelines. The Organization of Persons with Psychosocial Disabilities (Porque) was engaged in community-building initiatives and combating stigma through public relations and support activities. For example, they published newspaper articles and appeared on television to raise awareness. Other organizations demonstrated limited activity with the least number of activities. Overall, the more active organizations prioritized policy, research, and communication, while activities related to community support and education were less frequent, indicating potential areas for development.

## Discussion

This review identified various psychiatric burdens in Japan, including epidemiological, clinical management, societal welfare, humanistic, and economic perspectives. Although some stakeholder activities address these challenges, many gaps still remain.

Based on the characteristics of the included studies, despite the clinical and societal importance of schizophrenia, Japanese literature remains limited. Notably, publications declined over the past decade, suggesting reduced research attention. While clinical management and epidemiology were well represented, humanistic, societal, and economic burdens were underreported. Similarly, gray literature, including conference abstracts and other sources, was not plentiful. These trends highlight the need for renewed focus and sustained research efforts to capture the multifaceted burden of schizophrenia in Japan.

Schizophrenia’s prevalence in Japan (0.6%^5^) is comparable to global rates (0.3–0.6%^2,63–65^). Additionally, a systematic review by Saha et al. demonstrated that the mortality gap between schizophrenia patients and the general population is worsening globally, underscoring the need for targeted interventions^66^. Reported mortality data^11–15^ appears higher than the global average (~2.5%^67^), possibly due to study populations with comorbid conditions such as cancer and AMI, or emergency admissions. These patients have a higher mortality risk compared to general schizophrenia patients. Comorbidities, such as hypertension, diabetes, and depression, further complicate health and contribute to disease burden^16–18^. In Australia, national surveys by Morgan et al. emphasized the importance of integrating mental health services with physical healthcare to address comorbidities and improve patient outcomes^68^. To address these issues in Japan, more epidemiological research is needed to inform public health strategies, early interventions, and equitable healthcare policies.

In addition to physical health issues, suicide is a major contributor to mortality in schizophrenia. Several studies in this review addressed suicide and suicide attempts, underscoring the importance of suicide prevention in this population. While motives were not detailed in the reviewed literature, previous research has identified hallucinations and delusions as common triggers, along with social factors such as living alone and unemployment^69^. These findings highlight the need for both clinical and social support strategies to reduce excess mortality.

Clinical management of schizophrenia presents challenges due to the complex treatment regimens, long-term hospitalization, and medication side effects. Functional impairments, such as those related to ADLs and cognitive dysfunction, also added to the clinical management burden. However, stakeholder activities targeting these were limited in this review. Holistic care and expanded insurance coverage for cognitive assessments and counseling are needed to close these gaps. Clinical guidelines help standardize care, but implementation remains inconsistent. This is a global issue—despite their benefits, full adoption of guidelines is still challenging in the US and Europe^3,70,71^. The search of stakeholders’ activities revealed that, in Japan, the EGUIDE project has promoted guideline use by training psychiatrists. A multi-center study showed higher rates of key prescribing indicators, suggesting education improves practice^72^. Ongoing efforts like EGUIDE are essential to strengthen care quality.

The COVID-19 pandemic had measurable impacts on individuals with schizophrenia, as shown in gray literature. These included increased self-harm and suicidal ideation, shorter hospital stays, and an increase in discharge cases. Such findings highlight the vulnerability of this population during public health crises. To mitigate these effects, healthcare service and policy adaptations are essential. In Japan, early intervention of healthcare services for psychosis is still developing, with efforts focused on reducing the duration of untreated psychosis and building community-based networks for early detection and support^73^. Additionally, telemedicine has shown promise in improving treatment adherence and reducing relapse rates among patients with schizophrenia, especially during periods of restricted access to in-person care^74^. Strengthening these healthcare services could help maintain continuity of care and reduce the burden on patients, caregivers, and healthcare systems during future crises.

The stigma influences patients’ willingness to seek treatment and social integration, hindering access to public assistance and increasing emotional and social toll. Welfare support was insufficient, with stakeholders providing additional support. Although stigma is a global issue, New Zealand progressed through nationwide anti-stigma campaigns like “Like Minds, Like Mine,” which could guide Japan^75^. In Japan, patient organizations advocate through media or events. Kutcher et al. suggested stigma reduction requires context-specific, developmentally appropriate mental health literacy integrated into schools and communities^76^. Adopting similar strategies in Japan could help improve care access and welfare services.

Strengthening caregiver support systems and ensuring healthcare continuity during crises are essential. The review found that family members bear a disproportionate caregiving burden, risking mental health issues. Stakeholders’ activities were identified to address these challenges. According to Chien and Norman, mutual support groups for caregivers have been shown to reduce stress and improve coping mechanisms^77^. However, in the review, the burden during disasters/pandemics was identified but not addressed by stakeholder activities. The caregiver’s burden is especially relevant given the earlier discussion on COVID-19’s impact on patients, which likely also intensified the caregiving burden on families. Community-based disaster preparedness programs in the Philippines implemented programs that specifically address vulnerable populations^78^, could be adapted to Japan’s disaster-prone environment.

The economic burden of schizophrenia is substantial. Indirect costs, such as productivity loss from hospitalization or outpatient visits, and future income lost from premature death, exceed direct costs^59^. Despite this, our review did not identify any stakeholder activities addressing productivity losses or healthcare-related costs. Mitigating economic burden requires sustainable and comprehensive healthcare systems with public funding and insurance. Assessment of national and regional healthcare cost management is critical, as in the US case, where the economic burden of schizophrenia has been extensively documented, with estimates including both direct healthcare costs and indirect societal costs such as lost productivity^79,80^.

### Limitations and strengths of research

This review leverages gray literature, often excluded from traditional literature reviews, thereby adding unique value by capturing a broader range of real-world evidence and activities. While hand searches for gray literature may introduce bias or overlook less publicized initiatives, this approach ensures a more comprehensive understanding of the current landscape. Although limited to 2013–2023, focusing on recent developments ensures that the findings are highly relevant to current healthcare policies. The research does not aim to evaluate the effectiveness of interventions by stakeholders’ activities for schizophrenia but instead highlights existing initiatives and identifies gaps between stakeholders’ activities and unmet needs, serving as a foundation for future intervention studies. Further research with broader methodologies like implementation science is necessary for a comprehensive understanding of patient circumstances and healthcare policies, but this review provides critical groundwork for future studies to address these gaps.

## Conclusions

This review revealed the multifaceted and complex burden of schizophrenia from epidemiological, clinical, societal, welfare, humanistic, and economic perspectives. Policy recommendations include enhancing early diagnosis, standardizing treatment protocols, increasing public health funding, and improving welfare support systems to reduce the overall burden of schizophrenia. Effective strategies require collaborative approaches, including community-integrated care frameworks, multidisciplinary teams, and inclusive social welfare systems. However, the limited evidence underscores the need for evidence-based strategies, requiring further research, such as longitudinal studies and real-world data analyses, to monitor trends and evaluate healthcare interventions. These efforts will ensure that future strategies are grounded in robust evidence and tailored to the needs of patients and caregivers.

## Supplementary information

Table S1, Table S2, and Document S1

## Data Availability

Minimal data needed to interpret, replicate, or build upon findings can be made available upon reasonable request to the corresponding author.
