# Supplementary material for: Clinical, social, and economic burdens of schizophrenia in Japan: a targeted literature review
Source: Schizophrenia (Heidelb). 2026 Jan 20;12(1):27. doi: 10.1038/s41537-025-00716-9 (PMC12949227; doi:10.1038/s41537-025-00716-9)
Supplement: Supplementary file 1 — Table S1, Table S2, and Document S1 [file 41537_2025_716_MOESM1_ESM.docx]

**Supplementary Table S1. Data Sources for Grey Literature and Stakeholders’ Activities**

| **No.** | **Data sources names (only Japan-based organizations)** |
| --- | --- |
| 1 | Japanese Society of Schizophrenia Research |
| 2 | Japanese Society of Psychiatry and Neurology |
| 3 | Japan Academy of Psychiatric and Mental Health Nursing |
| 4 | Japanese Society for Social Psychiatry |
| 5 | Japanese Society of Neuropsychopharmacology |
| 6 | Japanese Association for Emergency Psychiatry |
| 7 | National Center of Neurology and Psychiatry |
| 8 | Evidence-based Information site on community lives for people with mental illness |
| 9 | Health and Global Policy Institute |
| 10 | Ministry of Health, Labor, and Welfare (MHLW) |
| 11 | The National Federation of Associations of Families with Mental Illness in Japan |
| 12 | Porque (Organization of Persons with Psychosocial Disabilities) |
| 13 | COMHBO (Community Mental Health and Welfare Organization) |
| 14 | Smile Navigator |
| 15 | Kokoro-share |

**Supplementary Table S2. Representative Search Strategy**

| **Set** | **Search key terms** |
| --- | --- |
| #1 | Schizophrenia[TIAB] or “Schizophrenia”[MeSH] |
| #2 | Japan*[TW] AND (patient*[TIAB] OR caregiver*[TIAB] OR carer*[TIAB]) |
| #3 | #1 AND #2 |
| Outcomes | |
| #4 | "Incidence"[MeSH] OR inciden*[TIAB] OR "Prevalence"[MeSH] OR prevalen*[TIAB] OR risk[TIAB] OR "Morbidity"[MeSH] OR morbidity[TIAB] OR comorbid*[TIAB] OR complication*[TIAB] OR "Mortality"[MeSH] OR mortal*[TIAB] OR "Death"[MeSH] OR death*[TIAB] OR died[TIAB] OR diabetes[TIAB] OR suicide[TIAB] |
| #5 | ((Treatment*[TIAB] or therap*[TIAB]) AND (option[TIAB] OR outcome[TIAB] OR prognosis[TIAB] OR comorbidities[TIAB])) OR “nursing care”[TIAB] OR (management[TIAB] AND comorbidities[TIAB]) OR "standard of care"[TIAB] OR SoC[TIAB] OR "healthcare resource*"[TIAB] OR "health care resource*"[TIAB] OR "healthcare utili*"[TIAB] OR outpatient*[TIAB] OR "hospitalization"[MeSH] OR hospital*[TIAB] OR "Inpatients"[MeSH] OR inpatient*[TIAB] OR (chronic[TIAB] AND management[TIAB]) OR (prevention [TIAB] AND relapse[TIAB]) OR “activities of daily living”[TIAB] OR ADL[TIAB] OR ((Social*[TIAB] OR capacity[TIAB] OR Daily[TIAB] OR “Real world”[TIAB] OR Real-world[TIAB]) AND function[TIAB]) |
| #6 | (“Social welfare”[TIAB] AND service[TIAB]) OR “community medicine”[TIAB] OR stigma[TIAB] OR awareness[TIAB] OR “healthcare policy”[TIAB] OR “health policy”[TIAB] OR “multidisciplinary team”[TIAB] OR MDT[TIAB] OR ((multidisciplinary[TIAB] OR work[TIAB] OR employ*[TIAB] OR housing[TIAB] OR financ*[TIAB]) AND support[TIAB]) OR “peer support”[TIAB] OR (accessibility[TIAB] AND treatment[TIAB] AND “mental health”[TIAB]) |
| #7 | (burden[TIAB] AND (disease[TIAB] OR treatment[TIAB])) OR "Quality of Life"[MeSH] OR "quality of life"[TIAB] OR QOL[TIAB] OR "health-related quality of life"[TIAB] OR HRQOL[TIAB] OR utilit*[TIAB] OR "quality-adjusted life year*"[TIAB] OR "Quality-Adjusted Life Years"[MeSH] OR QALY[TIAB] OR "health status"[TIAB] OR "patient reported outcome*"[TIAB] OR (cognitive[TIAB] AND impairment[TIAB]) OR "disability-adjusted life year"[TIAB] OR DALY[TIAB] OR (recovery[TIAB] AND (social[TIAB] OR personal[TIAB])) OR "Caregiver Burden"[MeSH] OR "Caregiver Burden"[TIAB] |
| #8 | cost*[TIAB] or economic*[TIAB] OR expenditure*[TIAB] OR budget*[TIAB] OR productivity[TIAB] OR cost-effectiv*[TIAB] OR cost-utility[TIAB] OR ICER[TIAB] OR "models, economic"[MeSH] OR "Costs and cost analysis"[MeSH] OR "Cost-benefit analysis"[MeSH] OR cost-benefit*[TIAB] OR "Cost of illness"[MeSH] OR "Health care costs"[MeSH] OR "Health expenditures"[MeSH] OR "absenteeism"[MeSH] OR absenteeism[TIAB] OR unemploy*[TIAB] OR WPAI[TIAB] OR “societal perspective”[TIAB] OR presenteeism[TIAB] OR ("work"[TIAB] AND "loss"[TIAB]) OR ("work"[TIAB] AND "disability"[TIAB]) |
| #9 | (#4 OR #5 OR #6 OR #7 OR #8) |
| Filters and Combinations | |
| #10 | (English[LA] OR Japanese[LA]) AND ("2013"[PDAT] : "2023"[PDAT]) NOT ((Clinical trial[PT] OR Editorial[PT] OR Comment[PT] OR "Case Reports"[PT]) OR in-vitro[MeSH] OR (Animals[Mesh] NOT (Humans[MeSH] AND Animals[MeSH]))) |
| #11 | #3 AND #9 AND #10 |

**Supplementary Document S1. List of All Extracted References Excluding Those Listed in the Main Text**

1. Tsukahara M, So R, Yosomihimura Y, Yamashita R, Yada Y, Kodama M, et al. Effect of smoking habits and concomitant valproic acid use on relapse in patients with treatment-resistant schizophrenia receiving clozapine: A 1-year retrospective cohort study. Acta Psychiatr Scand. 2023 Nov;148(5):437-446.
2. "Iwasaki Y, Yamasaki F. Involvement of Home Healthcare Nurses in Respite Hospitalization as a Form of Community Living Support for Clients with Schizophrenia. Journal of Japan Society of Nursing Research. 2023 Apr 20;46(1):1_33-1_42 (In Japanese).
3. Sato Y, Sugawara N, Kawamata Y, Shimoda K, Yasui-Furukori N. Survey of Health Literacy Among Japanese Outpatients with Mental Illness. Neuropsychiatr Dis Treat. 2023 Jun 2;19:1369-1378.
4. Arai Y, Sasayama D, Kuraishi A, Sahara R, Murata S, Tanaka A, et al. Sodium Valproate Use in Japanese Patients with Schizophrenia and Coronavirus Disease Is Associated with an Increased Risk of Pneumonia. J Clin Med. 2023 Sep 13;12(18):5953.
5. Yasui-Furukori N, Kawamata Y, Sasaki T, Yokoyama S, Okayasu H, Shinozaki M, et al. Prescribing Trends for the Same Patients with Schizophrenia Over 20 Years. Neuropsychiatr Dis Treat. 2023 Apr 17;19:921-928.
6. Onitsuka T, Okada T, Hasegawa N, Tsuboi T, Iga JI, Yasui-Furukori N, et al. Combination Psychotropic Use for Schizophrenia With Long-Acting Injectable Antipsychotics and Oral Antipsychotics: A Nationwide Real-World Study in Japan. J Clin Psychopharmacol. 2023 Jul-Aug 01;43(4):365-368.
7. Tsuboi F, Miyano K, Kashiwaba H, Saito F, Fujii H. Difficulties brought to families with schizophrenia patients who have experienced the tsunami caused by the Great East Japan Earthquake: From the viewpoint of the initial period of the disaster and the reconstruction period. Journal of Japan Academy of Human Care Science. 2023;16(2):1–11 (In Japanese).
8. Hayakawa K, Watabe M, Horikawa H, Sato-Kasai M, Shimokawa N, Nakao T, et al. Low-Density Lipoprotein Cholesterol Is a Possible Blood Biomarker of Schizoid Personality Traits among Females. J Pers Med. 2022 Jan 19;12(2):131.
9. Watanabe Y, Ono S, Sugai T, Suzuki Y, Yamazaki M, Sugawara N, et al. Associations between the number of antipsychotics prescribed and metabolic parameters in Japanese patients with schizophrenia. PCN Rep. 2022 Jul 7;1(3):e28.
10. "Hagane Y, Kiuchi S, Ihara H, Shioya N, Hashizume N. Clinical nutritional studies on atherosclerosis risk factors in hospitalized patients with schizophrenia. New diet therapy. 2023;39(3):3–17 (In Japanese).
11. Nakagawa Y, Kumoi H, Sasaki H, Yamada S. Positional Stability of the Non-Dominant Hand is Associated with Difficulties in Daily Functioning in Schizophrenia. Asian Journal of Occupational Therapy. 2023;19(1):124–31.
12. Higuchi S, Sako A, Kondo T, Kusanishi S, Enomoto T, Hayakawa T, et al. Clozapine Use in Japan Based on National Database of Health Insurance Claims and Specific Health Checkups Open Data: Disparities by Region, Age, and Sex. Psychiatria et neurologia Japonica. 2022;124(1):3–15 (In Japanese).
13. Okada H. Association of negative symptom domains and other clinical characteristics of schizophrenia on long-term hospitalization. Indian J Psychiatry. 2022 May-Jun;64(3):277-283.
14. Shimada T, Kobayashi G, Saeki Y, Mizukoshi C, Chikazawa K, Nokura K, et al. A Retrospective Study on the Relationship Between Cognitive Function and Social Function in Patients With Schizophrenia. J Clin Med Res. 2022 Sep;14(9):348-356.
15. Uchino T, Okubo R, Takubo Y, Aoki A, Wada I, Hashimoto N, et al. Perceptions of and subjective difficulties with social cognition in schizophrenia from an internet survey: Knowledge, clinical experiences, and awareness of association with social functioning. Psychiatry Clin Neurosci. 2022 Sep;76(9):429-436.
16. Konishi T, Fujiogi M, Michihata N, Tanaka-Mizutani H, Morita K, Matsui H, et al. Breast cancer surgery in patients with schizophrenia: short-term outcomes from a nationwide cohort. Br J Surg. 2021 Mar 12;108(2):168-173.
17. Hori H, Atake K, Katsuki A, Yoshimura R. Effects of the number of hospitalizations on cognitive function in Japanese patients with stable schizophrenia. CNS Spectr. 2021 Dec 4;26(6):658-663. doi: 10.1017/S1092852920001728.
18. Kitamura N, Shigiyama T, Murao T, Ebihara K, Doihara K, Yabe T, et al. Reduction of admission rate after standardized family psychoeducational program: An assessment based on the Family Attitude Scale | Article Information | J-GLOBAL. Kawasaki Medical Journal. 2021;47:49–59 (In Japanese).
19. Numata Y, Nanba M. Assessment Particulars for Judging the Possibility of Discharge for Long-Term Hospitalized Patients with Schizophrenia by Psychiatric Ward Nurses. Journal of Japan Academy of Psychiatric and Mental Health Nursing. 2022 Jun 30;31(1):1–9 (In Japanese).
20. Maki S, Nagai K, Ando S, Tamakoshi K. Structure and predictors of in-hospital nursing care leading to reduction in early readmission among patients with schizophrenia in Japan: A cross-sectional study. PLoS One. 2021 Apr 30;16(4):e0250771.
21. Usami S, Nakayama Y, Nozue K, Fujii M, Ooi M. The Nursing Care for Psychiatric Patients to Prevent Hospital Readmission. Journal of Japan Academy of Psychiatric and Mental Health Nursing. 2014 Jun 20;23(1):70–80 (In Japanese).
22. Toyoda K, Hata T, Yamauchi S, Kinoshita S, Nishihara M, Uchiyama K, et al. A descriptive study of 10-year clozapine use from the nationwide database in Japan. Psychiatry Res. 2021 Mar;297:113764.
23. Imazu S, Hata T, Toyoda K, Kubo Y, Yamauchi S, Kinoshita S, et al. Safety profile of clozapine: Analysis using national registry data in Japan. J Psychiatr Res. 2021 Sep;141:116-123.
24. Inagaki A, Sato H, Inada K, Ichihashi K, Nakagawa A, Furukoori N, et al. Safety of antipsychotic pharmacotherapy for schizophrenia: a review of clinical trials and post-marketing surveillance studies conducted in Japan. Japanese journal of clinical psychopharmacology. 2021 Nov;24(11):1153–69 (In Japanese).
25. Sato K, Yoshimura B, Ishigami H, Hatsutori T, Murao T, Kishi Y. Comprehensive care for first-episode schizophrenia. The continuity from inpatient psychiatric treatment to community care. Japanese Journal of Preventive Psychiatry. 2016;1(1):68–79 (In Japanese).
26. Ichinose M, Miura I, Horikoshi S, Yamamoto S, Kanno-Nozaki K, Watanabe K, et al. Effect of Switching to Brexpiprazole on Plasma Homovanillic Acid Levels and Antipsychotic-Related Side Effects in Patients with Schizophrenia or Schizoaffective Disorder. Neuropsychiatr Dis Treat. 2021 Apr 13;17:1047-1053.
27. Koreki A, Mori H, Nozaki S, Koizumi T, Suzuki H, Onaya M. Risk of Nonalcoholic Fatty Liver Disease in Patients With Schizophrenia Treated With Antipsychotic Drugs: A Cross-sectional Study. J Clin Psychopharmacol. 2021 Jul-Aug 01;41(4):474-477.
28. Matsuzaki H, Hatano M, Iwata M, Yamada S. Treatment Continuation of Asenapine or Olanzapine in Japanese Schizophrenia Patients: A Propensity Score Matched Study. Neuropsychiatr Dis Treat. 2021 Dec 14;17:3655-3661.
29. Yasuma N, Yamaguchi S, Ogawa M, Shiozawa T, Abe M, Igarashi M, et al. Care difficulties and burden during COVID-19 pandemic lockdowns among caregivers of people with schizophrenia: A cross-sectional study. Neuropsychopharmacol Rep. 2021 Jun;41(2):242-247.
30. Nagata K, Kitaoka K, Kawamura M. Experiences and perceptions of people living with schizophrenia in Japan: A qualitative study. Nurs Health Sci. 2021 Dec;23(4):782-791.
31. Uju Y, Kanzaki T, Yamasaki Y, Kondo T, Nanasawa H, Takeuchi Y, et al. Metabolic changes of Japanese schizophrenic patients transferred from hospitalization to outpatients. Glob Health Med. 2020 Jun 30;2(3):178-183.
32. Uju Y, Kanzaki T, Yamasaki Y, Kondo T, Nanasawa H, Takeuchi Y, et al. A cross-sectional study on metabolic similarities and differences between inpatients with schizophrenia and those with mood disorders. Ann Gen Psychiatry. 2020 Sep 22;19:53.
33. Takahashi T, Yamazaki I, Harada Y, Osada R, Yui S, Tanabe H, et al. Case reports of Cotard's syndrome in Japan: a review. Psychogeriatrics. 2020 Jul;20(4):540-541.
34. Nishio Y, Watanabe H, Miyaue T, Murakami T, Matsuda Y. Factors correlated with low body weight in elderly patients hospitalized in a psychiatric ward. Journal of the Japan Dietetic Association. 2020;63(11):617–26 (In Japanese).
35. Inoue K, Otsuka K, Onishi H, Cho Y, Shiraishi M, Narita K, et al. Multi-institutional survey of suicide death among inpatients with schizophrenia in comparison with depression. Asian J Psychiatr. 2020 Feb;48:101908.
36. Takahashi K, Yamazawa R, Suzuki T, Mimura M, Uchida H. Gap between patients with schizophrenia and their psychiatrists on the needs to psychopharmacological treatment: A cross-sectional study. Neuropsychopharmacol Rep. 2020 Sep;40(3):232-238.
37. Ogawa K, Mori C. Factors Related to the Meaning of Life in Patients Hospitalized with Schizophrenia. Journal of Japan Society of Nursing Research. 2022 Jan 20;44(5):5_709-5_719 (In Japanese).
38. Ichihashi K, Hori H, Hasegawa N, Yasuda Y, Yamamoto T, Tsuboi T, et al. Prescription patterns in patients with schizophrenia in Japan: First-quality indicator data from the survey of "Effectiveness of Guidelines for Dissemination and Education in psychiatric treatment (EGUIDE)" project. Neuropsychopharmacol Rep. 2020 Sep;40(3):281-286.
39. Hayashi R, Nagai A, Shikai M, Kuroda K, Tanaka H, Inadomi H. Metacognitive Training for Patients with Schizophrenia Improves Cognitive Insights. Journal of Rehabilitation and Health Sciences. 2020;16:7–10 (In Japanese).
40. Iwata N, Inagaki A, Sano H, Niidome K, Kojima Y, Yamada S. Treatment Persistence Between Long-Acting Injectable Versus Orally Administered Aripiprazole Among Patients with Schizophrenia in a Real-World Clinical Setting in Japan. Adv Ther. 2020 Jul;37(7):3324-3336.
41. Kamei H, Homma Y, Takeuchi I, Hajitsu G, Tozawa K, Hatano M, et al. Acceptance of the Deltoid Muscle Injection of Aripiprazole Long-acting Injectable in the Patients with Schizophrenia. Clin Psychopharmacol Neurosci. 2020 Feb 29;18(1):49-57.
42. Iwamoto Y, Fujino N. The Viewpoints of Psychiatric Nurses That Are Necessary to Identify Suicide Risk among Inpatients with Chronic-Stage Schizophrenia. Journal of Japan Academy of Psychiatric and Mental Health Nursing. 2020 Jun 30;29(1):60–9 (In Japanese).
43. Yoshida N, Kageyama M. Experiences of Individuals Schizophrenia Who Were Violent against Their Parents. Japanese Journal of Public Health Nursing. 2020;9(2):81–90 (In Japanese).
44. Shimada T, Kobayashi M, Ohori M, Inagaki Y, Shimooka Y, Ishihara I. Cost-Effectiveness of Individualized Occupational Therapy for Schizophrenia: Results from a Two-Year Randomized Controlled Trial. Asian Journal of Occupational Therapy. 2020;16(1):29–34.
45. Kasahara-Kiritani M, Chaturvedi A, Inagaki A, Wakamatsu A, Jung W. Budget impact analysis of long acting injection for schizophrenia in Japan. J Med Econ. 2020 Aug;23(8):848-855.
46. Toriyama K, Mikami T, Sato Y, Sasajima T. Post marketing survey of clozapine (Clozaril) in Japanese patients of treatment resistant schizophrenia (final report). Japanese Journal of Clinical Psychopharmacology. 2019;22(11):1107–39 (In Japanese).
47. Kaneko F, Okamura H. Discrepancies between Self- and Clinical Staff Members' Perception of Cognitive Functioning among Patients with Schizophrenia Undergoing Long-Term Hospitalization. Occup Ther Int. 2019 Nov 3;2019:6547096. doi: 10.1155/2019/6547096. PMID: 31777475; PMCID: PMC6875358.
48. Nakamura Y, Shibata I, Mahlich J. Modeling the Choice Between Risperidone Long-Acting Injectable and Generic Risperidone from the Perspective of a Japanese Hospital. Neurol Ther. 2019 Dec;8(2):433-447. doi: 10.1007/s40120-019-0147-y. Epub 2019 Aug 10. PMID: 31401796; PMCID: PMC6858920.
49. Doi N, Iizuka S, Omi A, Koyanagi M, Kikuchi J, Akiyama S. Influence of the Medical Fee Revision on Type of Antipsychotic Prescriptions by Physicians for Patients with Schizophrenia. Applied Therapeutics. 2019;13:16–26 (In Japanese).
50. Shiozawa T, Matsunaga A, Sato S, Fujii C. Comparison of information requested by family members of schizophrenic patients and information provided by psychiatric visiting nursing staffs: The questionnaire survey via the Internet. Japanese Journal of Clinical Psychiatry. 2019;48(7):887–98 (In Japanese).
51. Araki T, Urizaki T, Yamauchi S, Komatsu H. Examination of Related Factors of Nurses’ Attitudes toward Terminal Care of Schizophrenic Patients in Psychiatric Hospitals in Japan. Journal of Japan Academy of Psychiatric and Mental Health Nursing. 2019 Nov 30;28(2):57–68 (In Japanese).
52. Watanabe S, Sugihara M. Long-term hospitalization among patients with schizophrenia: Influence of Defeatist Beliefs, Negative Symptoms and individual factors on discharge intention. Japanese Occupational Therapy Research. 2019 Jun 15;38(3):314–24 (In Japanese).
53. Sugawara N, Maruo K, Sugai T, Suzuki Y, Ozeki Y, Shimoda K, et al. Prevalence of underweight in patients with schizophrenia: A meta-analysis. Schizophr Res. 2018 May;195:67-73.
54. Tanimoto S, Yayama S, Suto S, Matoba K, Kajiwara T, Inoue M, et al. Self-harm and Suicide Attempts in a Japanese Psychiatric Hospital. East Asian Arch Psychiatry. 2018 Mar;28(1):23-27.
55. Ebisu T, Sawada T, Kawashima Y, Hashimoto T, Yotsumoto K. Relationship between functioning and psychiatric symptoms in long-term inpatients with schizophrenia. Japanese Occupational Therapy Research. 2018;37(3):295–300 (In Japanese).
56. Matsumoto Y, Okimoto K, Watanabe K. Process of Perspective-Taking by Expert Nurses of Schizophrenic Patients with Unacceptable Behavior. Journal of Japan Academy of Psychiatric and Mental Health Nursing. 2018 Jun 30;27(1):9–20 (In Japanese).
57. Inada K, Oshibuchi H, Ishigooka J, Nishimura K. Analysis of Clozapine Use and Safety by Using Comprehensive National Data From the Japanese Clozapine Patient Monitoring Service. J Clin Psychopharmacol. 2018 Aug;38(4):302-306.
58. "Maki S, Nagai K, Ando S. The Process of the Support by the Proficient and Expert Hospital Nurses for the Community Settlement of the Discharged Schizophrenia Patients Who Were Readmitted within Three Months. Journal of Japan Society of Nursing Research. 2018 Sep 20;41(4):4_713-4_722 (In Japanese).
59. Matsumoto K, Ueno M, Okawa S. Public Health Nurses’ Support Based on the Perspective of the Viability among Residents with Schizophrenia Who Are Untreated or with Interrupted Treatment. Journal of Japan Academy of Community Health Nursing. 2018;21(2):31–9 (In Japanese).
60. Fujino H, Sumiyoshi C, Yasuda Y, Yamamori H, Fujimoto M, Fukunaga M, et al. Frontiers in Psychiatry: Estimating Cognitive Impairment in Patients with Schizophrenia – A Multicenter Collaborative Study. Psychiatria et neurologia Japonica. 2018;120(4):255–61 (In Japanese).
61. Kageyama M, Solomon P, Yokoyama K, Nakamura Y, Kobayashi S, Fujii C. Violence Towards Family Caregivers by Their Relative with Schizophrenia in Japan. Psychiatr Q. 2018 Jun;89(2):329-340 (In Japanese).
62. Kageyama M, Solomon P. Post-traumatic stress disorder in parents of patients with schizophrenia following familial violence. PLoS One. 2018 Jun 1;13(6):e0198164.
63. Kasuga A, Shimizu K. Experiences of Community: Dwelling Schizophrenics Who Use Psychiatric Day Care Services in Developing Life Prospects. Journal of Japan Academy of Psychiatric and Mental Health Nursing. 2018 Jun 30;27(1):63–74 (In Japanese).
64. Sugawara H, Mori C. The self-concept of person with chronic schizophrenia in Japan. Neuropsychopharmacol Rep. 2018 Sep;38(3):124-132.
65. Sruamsiri R, Mori Y, Mahlich J. Productivity loss of caregivers of schizophrenia patients: a cross-sectional survey in Japan. J Ment Health. 2018 Dec;27(6):583-587.
66. Omi T, Ito H, Riku K, Kanai K, Takada H, Fujimi S, et al. Possible factors influencing the duration of hospital stay in patients with psychiatric disorders attempting suicide by jumping. BMC Psychiatry. 2017 Mar 20;17(1):99.
67. Uchida S, Ichinose T, Iizuka Y, Okamura K, Shitara H, Yamazaki M, et al. Osteopenia and the physical function in Japanese patients with schizophrenia. Arch Osteoporos. 2017 Oct 27;12(1):93.
68. Hatano M, Kamei H, Kato A, Takeuchi I, Hanya M, Uno J, et al. Assessment of the Latent Adverse Events of Antipsychotic Treatment Using a Subjective Questionnaire in Japanese Patients with Schizophrenia. Clin Psychopharmacol Neurosci. 2017 May 31;15(2):132-137.
69. Miyauchi M, Kishida I, Suda A, Shiraishi Y, Fujibayashi M, Taguri M, et al. Long term effects of smoking cessation in hospitalized schizophrenia patients. BMC Psychiatry. 2017 Mar 7;17(1):87.
70. Narita T, Kobayashi K. Community Living Conditions of People Living with Schizophrenia in Japan: Focusing on the Background of the Continuance of Community Life. International Medical Journal. 2017;24(6):442–6.
71. Kiriyama K, Matsushita T. An Attempt In The Treatment Plan That Aimed At Ability For Self-Decision Improvement Of The Schizophrenia Patient : Patients Oneself Drafts A Treatment Plan With The Nurse And Performs It And Evaluates It. Japanese Journal of Study in Addiction Nursing. 2017 Feb 28;14(1):2–10 (In Japanese).
72. Setoguchi H, Itomine I. Trends in Research on Agreement with the Disease in Patients with Schizophrenia. JOURNAL OF THE JAPANESE ASSOCIATION OF RURAL MEDICINE. 2017 Jan 31;65(5):917–23 (In Japanese).
73. Fujimoto Y, Fujino Y, Matsuura E, Kusuba Y. Correlation Between the Recovery Level and Background Factors of Schizophrenics in the Community. JOURNAL OF JAPAN HEALTH MEDICINE ASSOCIATION. 2017 Feb 21;25(4):335–9 (In Japanese).
74. Sugawara N, Yasui-Furukori N, Yamazaki M, Shimoda K, Mori T, Sugai T, et al. Attitudes toward metabolic adverse events among patients with schizophrenia in Japan. Neuropsychiatr Dis Treat. 2016 Feb 24;12:427-36.
75. Tarutani S, Kikuyama H, Ohta M, Kanazawa T, Okamura T, Yoneda H. Association between Medication Adherence and Duration of Outpatient Treatment in Patients with Schizophrenia. Psychiatry Investig. 2016 Jul;13(4):413-9. doi: 10.4306/pi.2016.13.4.413. Epub 2016 Jul 25. PMID: 27482242; PMCID: PMC4965651.
76. Shimada T, Nishi A, Yoshida T, Tanaka S, Kobayashi M. Factors Influencing Rehospitalisation of Patients with Schizophrenia in Japan: A 1-year Longitudinal Study. Hong Kong J Occup Ther. 2016 Dec;28(1):7-14.
77. Takeuchi I, Hanya M, Uno J, Amano Y, Fukai K, Fujita K, et al. A Questionnaire-based Study of the Views of Schizophrenia Patients and Psychiatric Healthcare Professionals in Japan about the Side Effects of Clozapine. Clin Psychopharmacol Neurosci. 2016 Aug 31;14(3):286-94.
78. Akiyama K, Saito S, Saito A, Ozeki Y, Watanabe T, Fujii K, et al. Predictive value of premorbid IQ, negative symptoms, and age for cognitive and social functions in Japanese patients with schizophrenia: A study using the Japanese version of the Brief Assessment of Cognition in Schizophrenia. Psychiatry Res. 2016 Dec 30;246:663-671.
79. Hashimoto Y, Tensho M. The need for educating patients with schizophrenia about the adverse effects of medications. Australas Psychiatry. 2016 Aug;24(4):352-5.
80. Kageyama M, Solomon P, Kita S, Nagata S, Yokoyama K, Nakamura Y, et al. Factors related to physical violence experienced by parents of persons with schizophrenia in Japan. Psychiatry Res. 2016 Sep 30;243:439-45.
81. Iinuma M, Satodate E, Kawasaka H, Oikawa Y, Ogasawara R, Fujiwara K. Changes in Insight and Medication Awareness in Patients with Schizophrenia Through Psychoeducation. Nihon Kango Gakkai Ronbunshuu Seishin Kango. 2016;46:19–22 (In Japanese).
82. Kawabe K, Matsumoto S, Tada A, Mizuguchi K, Miki D, Horikawa K, et al. The brief assessment of cognition in chronic schizophrenia. Japanese Journal of Psychiatry. 2016;21(4):315–21 (In Japanese).
83. Hashimoto Y, Tensho M. Effect of pharmacist intervention on physician prescribing in patients with chronic schizophrenia: a descriptive pre/post study. BMC Health Serv Res. 2016 Apr 26;16:150.
84. Ito H, Kumagai T, Kimura M, Koike S, Shimizu T. Dietary Intake in Body Mass Index Differences in Community-Based Japanese Patients with Schizophrenia. Iran J Public Health. 2015 May;44(5):639-45.
85. Kanzaki T, Uju Y, Sekine K, Ishii Y, Yoshimi T, Yasui R, et al. Increased Silent Brain Infarction Accompanied With High Prevalence of Diabetes and Dyslipidemia in Psychiatric Inpatients: A Cross-Sectional Study. Prim Care Companion CNS Disord. 2015 Mar 26;17(2):10.4088/PCC.14m01713.
86. Sugai T, Suzuki Y, Yamazaki M, Shimoda K, Mori T, Ozeki Y, et al. High prevalence of underweight and undernutrition in Japanese inpatients with schizophrenia: a nationwide survey. BMJ Open. 2015 Dec 9;5(12):e008720.
87. Nakamura D, Takashio O, Iwanami A, Morita T, Ikeda A, Saitou K, et al. Reduced prevalence of cardiovascular disease and metabolic syndrome-related disorders among Japanese long-term inpatients with schizophrenia. Clinical Neuropsychopharmacology and Therapeutics. 2015;6(0):16–27.
88. Ikeda T, Makabe H. The Physical Functionality of Long-term Schizophrenic Inpatients at Our Hospital. Annual Report of The Tohoku Section of Japanese Physical Therapy Association. 2015;27:14–7 (In Japanese).
89. Tachimori H, Takeshima T, Kono T, Akazawa M, Zhao X. Statistical aspects of psychiatric inpatient care in Japan: Based on a comprehensive nationwide survey of psychiatric hospitals conducted from 1996 to 2012. Psychiatry Clin Neurosci. 2015 Sep;69(9):512-22.
90. Takahashi M, Nakahara N, Fujikoshi S, Iyo M. Remission, response, and relapse rates in patients with acute schizophrenia treated with olanzapine monotherapy or other atypical antipsychotic monotherapy: 12-month prospective observational study. Pragmat Obs Res. 2015 Jul 14;6:39-46.
91. Sumiyoshi C, Harvey PD, Takaki M, Okahisa Y, Sato T, Sora I, et al. Factors predicting work outcome in Japanese patients with schizophrenia: role of multiple functioning levels. Schizophr Res Cogn. 2015 Sep 9;2(3):105-112.
92. Sasamoto M, Okazaki A, Oinaka T, Kinmoto M. Learning contents of nurses in discharge support for patients with schizophrenia provided through inter-professional work at psychiatric hospitals. Japanese Red Cross Hiroshima Coll Nurs [Internet]. 2015 Mar 31 [cited 2025 Oct 15];15:21–9 (In Japanese).
93. Sasaki T, Yamada T. Self-recognition and discharge-recognition of patients hospitalized in psychiatric hospital. Japanese Journal of Occupational Behavior. 2015;19(3):151–60 (In Japanese).
94. Nagasawa K, Ishii N, Fujii S, Yuasa T. The relationship between rhythm synchronization and related factors in patients with schizophrenia. Bulletin of Graduate School of Health Sciences, Akita University. 2015;23(2):139–44 (In Japanese).
95. Imai A, Hayashi N, Shiina A, Sakikawa N, Igarashi Y. Factors associated with violence among Japanese patients with schizophrenia prior to psychiatric emergency hospitalization: a case-controlled study. Schizophr Res. 2014 Dec;160(1-3):27-32.
96. Sugawara N, Yasui-Furukori N, Sato Y, Saito M, Furukori H, Nakagami T, et al. Dietary patterns are associated with obesity in Japanese patients with schizophrenia. BMC Psychiatry. 2014 Jun 20;14:184. doi: 10.1186/1471-244X-14-184.
97. Suzuki Y, Sugai T, Fukui N, Watanabe J, Ono S, Tsuneyama N, et al. High prevalence of underweight and undernutrition in Japanese inpatients with schizophrenia. Psychiatry Clin Neurosci. 2014 Jan;68(1):78-82.
98. Harada K, Eto N, Honda Y, Kawano N, Ogushi Y, Matsuo M, et al. A comparison of the characteristics of suicide attempters with and without psychiatric consultation before their suicidal behaviours: a cross-sectional study. BMC Psychiatry. 2014 May 21;14:146.
99. Ikeshita K, Shimoda S, Norimoto K, Arita K, Shimamoto T, Murata K, et al. Profiling psychiatric inpatient suicide attempts in Japan. Int J Emerg Ment Health. 2014;16(1):217-21.
100. Ishii T, Hashimoto E, Ukai W, Kakutani Y, Sasaki R, Saito T. Characteristics of attempted suicide by patients with schizophrenia compared with those with mood disorders: a case-controlled study in northern Japan. PLoS One. 2014 May 8;9(5):e96272.
101. Teraishi T, Hori H, Sasayama D, Matsuo J, Ogawa S, Ishida I, et al. Relationship between lifetime suicide attempts and schizotypal traits in patients with schizophrenia. PLoS One. 2014 Sep 16;9(9):e107739.
102. Ochiai H, Otsubo T, Ikai H, Imanaka Y. Factors associated with high-dose prescription of antipsychotics in outpatients with schizophrenia── An analysis of claims data from a Japanese prefecture ──. Journal of the Japan Society for Healthcare Administration. 2014;51(4):183–91 (In Japanese).
103. Omiya H, Yamashita K, Miyata T, Hatakeyama Y, Yambe K, Matsumoto I. A practical study on Cognitive Remediation Therapy (CRT) using the Frontal/Executive Program (FEP) for patients with schizophrenia. Japanese journal of clinical psychiatry. 2014;43(10):1525–32 (In Japanese).
104. Inoue K, Inoue K, Suda S, Shioda K, Kobayashi T, Kishi K, et al. Functional impairment in outpatients with mental disorders after the 2011 Great East Japan Earthquake. Jichi Medical University Journal. 2014;36:47–55.
105. Nakamura H, Watanabe N, Matsushima E. Structural equation model of factors related to quality of life for community-dwelling schizophrenic patients in Japan. Int J Ment Health Syst. 2014 Jul 25;8:32.
106. Inamura Y, Sagae T, Kushida O, Nakamachi K, Murayama N. Survey of obesity and underweight among inpatients with schizophrenia in psychiatric hospitals throughout Japan. Seishin Shinkeigaku Zasshi. 2013;115(1):10-21.
107. Suzuki Y, Mikami T, Tajiri M, Kunizuka T, Abe H, Someya T. Effects of hospitalization in a psychiatric ward on the body weight of Japanese patients with schizophrenia. Int J Psychiatry Med. 2013;45(3):261-8.
108. Suzuki Y, Sugai T, Fukui N, Watanabe J, Ono S, Tsuneyama N, et al. Low prevalence of metabolic syndrome and its prediction in Japanese inpatients with schizophrenia. Hum Psychopharmacol. 2013 Mar;28(2):188-91.
109. Umene-Nakano W, Yoshimura R, Hoshuyama T, Yoshii C, Hayashi K, Nakano H, et al. Current smoking rate in patients with psychiatric disorders in Japan: questionnaire survey. Psychiatry Res. 2013 Nov 30;210(1):268-73.
110. Inamura Y. Preventing Physical Health Risks in Pharmacological Treatment of Schizophrenia: Health Issues in Patients with Schizophrenia from the Perspective of a Nutritionist. Japanese Journal of Clinical Psychopharmacology. 2013;16(5):675–81 (In Japanese).
111. Saito T, Koike S, Ozawa T, Usui Y. Risk Factors for Aspiration Pneumonia among Patients with Schizophrenia Suffering from Dysphagia. The Japanese Journal of Dysphagia Rehabilitation. 2013 Apr 30;17(1):52–9 (In Japanese).
112. Kimura R, Ikeda S, Kumazaki H, Yanagida M, Matsunaga H. Comparison of the clinical features of suicide attempters by jumping from a height and those by self-stabbing in Japan. J Affect Disord. 2013 Sep 5;150(2):695-8.
113. Tanioka T, Chiba S, Onishi Y, Kataoka M, Kawamura A, Tomotake M, et al. Factors associated with discharge of long-term inpatients with schizophrenia in Japan: a retrospective study. Issues Ment Health Nurs. 2013 Apr;34(4):256-64.
114. Sato M, Michigami K. Examining the Relationship Between the Level of Insight and Rehospitalization in Patients with Schizophrenia: An Evaluation Using the SAI-J. Nihon kango gakkai ronbunshu Seishin kango. 2013;43:90–2 (In Japanese).
115. Tamasato K. Significance of Attentive Listening to Chronic Schizophrenic Patients by Nurses and the Phases of Significance. Journal of Japan Academy of Psychiatric and Mental Health Nursing. 2013 Nov 30;22(2):58–67 (In Japanese).
116. Onose H, Shinohara Y, Kimura M. Consideration of Introducing the IMR Program for Inpatients with Chronic Schizophrenia: Reflections on Bidirectional Support with the Assigned Nurse. The Japanese Psychiatric Nursing Society. 2013;56(2):77–81 (In Japanese).
117. Takahashi M, Fujikoshi S, Nakahara N, Iyo M. The continuation rate of monotherapy with olanzapine or other antipsychotic drugs in patients with acute-phase schizophrenia ―A 1-year observational study in routine clinical practice―. Japanese Journal of Clinical Psychopharmacology. 2013;16(11):1649–60 (In Japanese).
118. Okumura Y, Noda T, Ito H. Antipsychotics prescribing patterns of patients with schizophrenia in Japan : Using the National Database of Health Insurance Claim Information and Specified Medical Checkups. Japanese Journal of Clinical Psychopharmacology. 2013;16(8):1201–15 (In Japanese).
119. Kurosawa M, Tensho M, Tanifuji H, Kato T, Uno J, Umeda K, et al. Prescription survey 2011 of inpatients with schizophrenia in Japan: examination about the new investigation items of BMI and abnormal ECG. Japanese Journal of Clinical Psychopharmacology. 2013;16(7):1041–50 (In Japanese).
120. Yoshimura K. Psychiatric Nurses’ “Barriers to Discharge Planning” toward Long-Term Schizophrenia Inpatients with a Plan about the Place of Living after the Patient’s Discharge from the Hospital: A Study of Psychiatric Nurses’ Attitudes. Journal of Japan Academy of Psychiatric and Mental Health Nursing. 2013 Jun 10;22(1):12–20 (In Japanese).
121. Iwasaki M, Mizuno E. Fathers of Adult Children with Schizophrenia : Paternal Coping and Attitudes toward the Child’s Disorder. Japan Health Medicine Association. 2013 Apr 30;22(1):36–42 (In Japanese).
122. Kagawa S, Nagoshi T, Awanou Y, Matuoka M, Minami T. Process of Nursing Practice among Expert Nurses in Relation to Discharge Support for Long-Term Patients with Schizophrenia. Journal of Japan Academy of Nursing Science. 2013 Mar 20;33(1):1_61-1_70 (In Japanese).
123. Fujita E, Kato D, Uchiyama S, Watanabe A, Takei H, Hoshi R, et al. Effectiveness of the Illness Management and Recovery Program for Patients with Schizophrenia. Clinical psychiatry. 2013;55(1):21–8 (In Japanese).
124. Koyama A. Process of self-determination of discharge after long-term hospitalization in patients with schizophrenia. Journal of Japanese Nursing Ethics. 2013 Mar 15;5(1):40–5 (In Japanese).
125. Kikuchi M, Iwase S, Nakatogawa S. Factors Promoting Care-Seeking behavior in People with Schizophrenia : A Qualitative Study at a Psychiatric Day-Care Facility. Journal of Japan Academy of Psychiatric and Mental Health Nursing. 2013 Nov 30;22(2):40–9 (In Japanese).
126. Kamei K, Ito T, Fujita K, Mori Y. Prevalence study of tardive dyskinesia at a university hospital. Proceedings of the Annual Meeting of the Japanese Society of Psychiatry and Neurology. 2023;119:414 (In Japanese).
127. Ida I, Yamasaki Y, Ishii A. Psychiatric Support for the Treatment of Physical Comorbidities in Patients with Schizophrenia at General Hospitals. Proceedings of the Annual Meeting of the Japanese Society of Psychiatry and Neurology. 2023;119:691 (In Japanese).
128. Mochizuki H, Okawa M, Uesato S, Nakamoto T. Nurses’ Involvement in Acute Psychiatric Wards to Promote a Sense of Security in Individuals with Schizophrenia. Proceedings of the Annual Conference of the Japanese Academy of Psychiatric and Mental Health Nursing. 2023;33:81 (In Japanese).
129. Narita Y, Watanabe R, Ishida K, Oguro S, Iwata T. Treatment Approaches for Catatonia in Schizophrenia: A Comparative Analysis of Prescriptions Between Catatonic and Non-Catatonic Groups. Proceedings of the Annual Meeting of the Japanese Society of Psychiatry and Neurology. 2023;119:415 (In Japanese).
130. Matsuda K, Hashimoto N. Treatment Outcomes of Long-Acting Injectable Antipsychotics and Post-Discontinuation Treatment Patterns: A Retrospective Investigation. Proceedings of the Annual Meeting of the Japanese Society of Psychiatry and Neurology. 2023;119:429 (In Japanese).
131. Takagi M, Katsuki F. Recovery of Parents with Children Diagnosed with Schizophrenia and Its Associated Factors. Proceedings of the Annual Conference of the Japanese Academy of Psychiatric and Mental Health Nursing. 2023;33:107 (In Japanese).
132. Watabe K. Relationship Between Expressed Emotion and Parental Distress Among Participants in Family Psychoeducation for Schizophrenia: Insights from Data Across Three Regions. Proceedings of the Annual Meeting of the Japanese Society of Psychiatry and Neurology. 2023;119:407 (In Japanese).
133. Nakai R, Kataoka M, Oe M. Loneliness Experienced by People with Schizophrenia Living in the Community with a History of Hospitalization. Proceedings of the Annual Conference of the Japanese Academy of Psychiatric and Mental Health Nursing. 2023;33:106 (In Japanese).
134. Tsutsumi Y, Tsugeno Y, Tatsuno G, Yamada R, Bun Y, Tanaka K, et al. Tardive Dyskinesia in Patients on Long-Term Antipsychotic Treatment. Proceedings of the Annual Meeting of the Japanese Society of Psychiatry and Neurology. 2022;118:521 (In Japanese).
135. Fukushima S, Matsumoto K. Report and Discussion of Outpatient and Inpatient Trends Before and After the COVID-19 Pandemic at Our Hospital. Proceedings of the Annual Meeting of the Japanese Society of Psychiatry and Neurology. 2022;118:639 (In Japanese).
136. Nakamura T, Furuhata R, Hasegawa N, Otsuki R, Furukoori N, Tsuboi T, et al. Factors Associated with Changes in Hypnotic Medication Use During Inpatient Treatment of Schizophrenia: Analysis of a Nationwide Survey. Proceedings of the Annual Meeting of the Japanese Society of Psychiatry and Neurology. 2022;118:518 (In Japanese).
137. Shimada T, Kobayashi G, Saeki Y, Mizukoshi C, Chikazawa K, Nokura K, et al. A Study on Factors Affecting Social Functioning in Schizophrenia. Proceedings of the Annual Meeting of the Japanese Society of Psychiatry and Neurology. 2022;118:545 (In Japanese).
138. Takemura Y, Okada S, Ishimaru A, Araga T, Matsuyama K, Yamashita M, et al. Consideration of the Indications for Modified Electroconvulsive Therapy in Elderly Patients with Hallucinations and Delusional States at Our Hospital. Proceedings of the Annual Meeting of the Japanese Society of Psychiatry and Neurology. 2022;118:644 (In Japanese).
139. Kobatake T, Matsumoto K, Konishi N, Kawamura K, Tokura E, Nishiumi R. A Literature Review on Self-Stigma and Coping Strategies Among Individuals with Schizophrenia. Proceedings of the Annual Conference of the Japanese Academy of Psychiatric and Mental Health Nursing. 2022;32:91 (In Japanese).
140. Hiyama R, Ohara I, Yoneyama N. Family Support Desired by Families of Patients with Schizophrenia in Medical Settings. Proceedings of the Annual Conference of the Japanese Academy of Psychiatric and Mental Health Nursing. 2022;32:159 (In Japanese).
141. Nishi M, Sugai T. Recovery of Individuals with Schizophrenia Using Home-Visit Nursing Services Across Life Stages. Proceedings of the Annual Conference of the Japanese Academy of Psychiatric and Mental Health Nursing. 2022;32:110 (In Japanese).
142. Fukuda D, Ikeuchi A, Mori C. Relationship Between Daily Life and Executive Function in Individuals with Chronic Schizophrenia. Proceedings of the Annual Conference of the Japanese Academy of Psychiatric and Mental Health Nursing. 2022;32:127 (In Japanese).
143. Suzuki M, Tanoue M, Mori C. Characteristics of Understanding Others’ Intentions and Their Relationship with Psychiatric Symptoms in Individuals with Schizophrenia: An Examination Using Hinting Tasks. Proceedings of the Annual Conference of the Japanese Academy of Psychiatric and Mental Health Nursing. 2022;32:132 (In Japanese).
144. Watanabe Y, Kanata S, Sato K, Inagaki A, Suga M, Sato S, et al. Factors Contributing to Social and Occupational Functioning in Schizophrenia: Associations with Cognitive Function and Psychiatric Symptoms. Proceedings of the Annual Meeting of the Japanese Society of Psychiatry and Neurology. 2022;118:545 (In Japanese).
145. Yokota H, Yamaguchi T, Kyo H, Nishida H, Hasegawa G. A Study on Obesity Among Long-Term Hospitalized Patients Receiving Antipsychotic Medication. Proceedings of the Annual Meeting of the Japanese Society of Psychiatry and Neurology. 2021;117:572 (In Japanese).
146. Hirota N, Kimoto S, Kishimoto N, Nishi Y, Honda M, Inoue K, et al. Clinical and demographic characteristics of the use of seclusion and restraint in NMU Psychiatric Institute (NMU-PI). Proceedings of the Annual Meeting of the Japanese Society of Psychiatry and Neurology. 2021;117:598 (In Japanese).
147. Ono M, Fujii K, Tsuji S, Yamazaki H, Murakami T, Ozeki Y. Evaluation of neuroleptic effect to the pregnant. Proceedings of the Annual Meeting of the Japanese Society of Psychiatry and Neurology. 2021;117:547 (In Japanese).
148. Suzuki R, Hasegawa M. The Process of Nursing Interventions Involved in Developing Crisis Plans for Discharge in Patients with Schizophrenia. Proceedings of the Annual Conference of the Japanese Academy of Psychiatric and Mental Health Nursing. 2021;31:103 (In Japanese).
149. Arai H, Hisamatsu M. Factors Influencing Changes in End-of-Life Care for Patients with Schizophrenia and Comorbid Cancer. Proceedings of the Annual Conference of the Japanese Academy of Psychiatric and Mental Health Nursing. 2021;31:147 (In Japanese).
150. Nishiyama H, Sugita H, Yamada H, Mera K, Tazaki T, Tomioka H, et al. A Study on Schizophrenia Patients Requiring Behavioral Restrictions in a Super-Acute Psychiatric Ward. Proceedings of the Annual Meeting of the Japanese Society of Psychiatry and Neurology. 2021;117:571 (In Japanese).
151. Inagawa M, Koizumi T, Minegishi S, Kusudo K, Onaya M, Koreki A. ognitive Rehabilitation Using Shogi in Schizophrenia: A Pre-Post Intervention Study. Proceedings of the Annual Meeting of the Japanese Society of Psychiatry and Neurology. 2021;117:570 (In Japanese).
152. Funai S, Tsuji T, Kusano E, Tabata M, Yamanoi R, Uenishi Y, et al. A Study on the Effectiveness of Psychoeducation in Patients with Schizophrenia. Proceedings of the Annual Meeting of the Japanese Society of Psychiatry and Neurology. 2021;117:577 (In Japanese).
153. Inuyama M, Machida Y, Imazu S, Nishizawa Y, Kinoshita S, Kanazawa T. Retrospective Study of 26 Patients Who Underwent Maintenance Electroconvulsive Therapy at Our Hospital. Proceedings of the Annual Meeting of the Japanese Society of Psychiatry and Neurology. 2021;117:632 (In Japanese).
154. Koyama Y, Yanagimoto K, Hakoda H, Oda K. The Relationship Between Pharmacotherapy and Quality of Life in Hospitalized Patients with Schizophrenia. Proceedings of the Annual Meeting of the Japanese Society of Psychiatry and Neurology. 2021;117:546 (In Japanese).
155. Ogata Y, Sugaya T, Mori C. Relationship Between Recovery, Self-Concept, and Self-Esteem in Individuals with Schizophrenia. Proceedings of the Annual Conference of the Japanese Academy of Psychiatric and Mental Health Nursing. 2021;31:142 (In Japanese).
156. Yamaguchi M, Fukuyama Y, Okamura K, Shimizu N. A Study on the Relationship Between Length of Hospitalization and Cognitive Function in Patients with Schizophrenia. Proceedings of the Annual Meeting of the Japanese Society of Psychiatry and Neurology. 2021;117:569 (In Japanese).
157. Ishii J, Kodaka F, Miyata H, Seto H, Inamura K, Nakamura S, et al. Association between functional prognosis and medication adherence in schizophrenia. Proceedings of the Annual Meeting of the Japanese Society of Psychiatry and Neurology. 2020;116:S.443 (In Japanese).
158. Akasaka K, Akasaka F, Akasaka T, Sadahiro S. Correlation Between Psychiatric Disorders and Bowel Conditions, Including Constipation, Among Psychiatric Outpatients. Proceedings of the Annual Meeting of the Japanese Society of Psychiatry and Neurology. 2020;116:569 (In Japanese).
159. Kobayashi N, Kikuchi S, Saito M, Honda N, Tomita H. Antipsychotic Treatment for Perinatal Schizophrenia and Associated Metabolic Complications. Proceedings of the Annual Meeting of the Japanese Society of Psychiatry and Neurology. 2020;116:441 (In Japanese).
160. Aoki N, Takekita Y, Kawashima H, Suwa T, Kinoshita T, Wada T. Survey Report on Continuation and Maintenance ECT in Japan. Proceedings of the Annual Meeting of the Japanese Society of Psychiatry and Neurology. 2020;116:460 (In Japanese).
161. Tanaka K. Mirror Image Study of Electroconvulsive Therapy (ECT) in Patients With Schizophrenia. Proceedings of the Annual Meeting of the Japanese Society of Psychiatry and Neurology. 2020;116:558 (In Japanese).
162. Kitamura H, Saito K, Munemura K, Takeda Y, Sato K, Tsurumaki M, et al. Prevalence of Sarcopenia Among Long-Term Psychiatric Inpatients. Proceedings of the Annual Meeting of the Japanese Society of Psychiatry and Neurology. 2019;115:442 (In Japanese).
163. Matsui K, Inada K, Ishibashi M, Kawano M, Oshibuchi H, Ishigooka J, et al. Agranulocytosis in Patients Treated With Clozapine-Findings From a Nationwide Survey in Japan-. Proceedings of the Annual Meeting of the Japanese Society of Psychiatry and Neurology. 2019;115:460 (In Japanese).
164. Onda K, Sugai T, Suzuki Y, Yamasaki M, Shimoda K, Mori T, et al. Differences in the Prevalence of Lifestyle-Related Diseases Between Outpatients and Inpatients With Schizophrenia. Proceedings of the Annual Meeting of the Japanese Society of Psychiatry and Neurology. 2019;115:471 (In Japanese).
165. Sugai T, Suzuki Y, Yamasaki M, Shimoda K, Mori T, Ozeki Y, et al. Relationship Between Health Awareness and Prevalence of MetS in Patients With Schizophrenia. Proceedings of the Annual Meeting of the Japanese Society of Psychiatry and Neurology. 2019;115:473 (In Japanese).
166. Takahashi Y, Hanaoka S, Abe T, Shibuya T, Fukami G, Hirata T. Forecast of Inpatient and Outpatient Numbers in the Psychiatric Emergency Medical Area of Chiba Prefecture. Proceedings of the Annual Meeting of the Japanese Society of Psychiatry and Neurology. 2019;115:608 (In Japanese).
167. Masaki K, Taniguchi N, Kamae K, Sudo Y, Tanaka H, Oya K, et al. Trends Among Elderly Involuntary Admissions in Psychiatric Emergency Wards (Super Emergency Units). Proceedings of the Annual Meeting of the Japanese Society of Psychiatry and Neurology. 2019;115:612 (In Japanese).
168. Fukuda K, Takemoto K, Ogawa H. Exploring Barriers to Discharge in Long-Term Psychiatric Hospitalization. Proceedings of the Annual Meeting of the Japanese Society of Psychiatry and Neurology. 2019;115:651 (In Japanese).
169. Suzuki Y, Sugai T, Yamasaki M, Shimoda K, Mori T, Ozeki Y, et al. Relationship Between Antipsychotic Polypharmacy and Tobacco Use Among Japanese Patients With Schizophrenia. Proceedings of the Annual Meeting of the Japanese Society of Psychiatry and Neurology. 2019;115:410 (In Japanese).
170. Fujiwara M, Higuchi Y, Inagaki M, Nakatani N, Fujimori M, Hayashibara C, et al. Smoking Cessation Stages and Subsequent Quitting Behavior in Outpatients With Schizophrenia: A Longitudinal Survey-Based Study. Proceedings of the Annual Meeting of the Japanese Society of Psychiatry and Neurology. 2019;115:436 (In Japanese).
171. Ono S, Sugai T, Suzuki Y, Yamasaki M, Shimoda K, Mori T, et al. Relationship Between Antipsychotic Medications and Triglyceride Levels in Japanese Patients With Schizophrenia: Differences Between Outpatients and Inpatients. Proceedings of the Annual Meeting of the Japanese Society of Psychiatry and Neurology. 2019;115:445 (In Japanese).
172. Otake M, Sugai T, Ono S, Suzuki Y, Yamasaki M, Shimoda K, et al. Differences in HDL Cholesterol Levels Among Schizophrenia Patients Treated With Different Antipsychotic Medications. Proceedings of the Annual Meeting of the Japanese Society of Psychiatry and Neurology. 2019;115:450 (In Japanese).
173. Arai H, Hisamatsu M. Factors Influencing Nurses’ Emotions When Providing End-of-Life Care to Patients with Schizophrenia and Comorbid Cancer. Proceedings of the Annual Conference of the Japanese Academy of Psychiatric and Mental Health Nursing. 2019;29:136 (In Japanese).
174. Ishii A. Nurses’ Experiences in Providing Postoperative Care to Patients with Schizophrenia and Characteristics of Their Postoperative Recovery. Proceedings of the Annual Conference of the Japanese Academy of Psychiatric and Mental Health Nursing. 2019;29:191 (In Japanese).
175. Kikuchi S, Kobayashi N, Honda N, Tomita H. A Retrospective Study on Pharmacotherapy and Relapse in Patients With Schizophrenia During the Perinatal Period. Proceedings of the Annual Meeting of the Japanese Society of Psychiatry and Neurology. 2019;115:443 (In Japanese).
176. Ozeki Y, Fujii K, Koelkebeck K, Shimoda K. Evaluation of the Impact of Comorbid Neurodevelopmental Disorders on the Clinical Course of Patients With Schizophrenia. Proceedings of the Annual Meeting of the Japanese Society of Psychiatry and Neurology. 2019;115:444 (In Japanese).
177. Watabe K. Changes in Parental Expressed Emotion Following Family Psychoeducation for Schizophrenia. Proceedings of the Annual Meeting of the Japanese Society of Psychiatry and Neurology. 2019;115:426 (In Japanese).
178. Naganuma H. Characteristics of Catatonic Schizophrenia. Proceedings of the Annual Meeting of the Japanese Society of Psychiatry and Neurology. 2019;115:443 (In Japanese).
179. Sakayori K, Yamamoto A, Masuoka T, Asayama K, Tateno A, Okubo Y. Current Status of Continuation and Maintenance Electroconvulsive Therapy at Nippon Medical School Hospital. Proceedings of the Annual Meeting of the Japanese Society of Psychiatry and Neurology. 2019;115:643 (In Japanese).
180. Kusuno Y. A Study on the Practice of Home-Based Psychiatric Care in Remote Areas. Proceedings of the Annual Meeting of the Japanese Society of Psychiatry and Neurology. 2019;115:772 (In Japanese).
181. Nemoto T, Aikawa S, Matsuo S, Mamiya N, Wada Y, Takubo Y, et al. Influence of Social Anxiety Symptoms on Social Functioning and Quality of Life in Individuals With Schizophrenia. Proceedings of the Annual Meeting of the Japanese Society of Psychiatry and Neurology. 2019;115:474 (In Japanese).
182. Imaeda E, Katsuki F. Examining the Factors Necessary for Community Living in Individuals with Schizophrenia: A Comparison Between Long-Term Hospitalized Patients and Daycare Users. Proceedings of the Annual Conference of the Japanese Academy of Psychiatric and Mental Health Nursing. 2019;29:148 (In Japanese).
183. Moriguchi M, Katsuki F. A Study on Self-Disclosure of Disability Among Outpatients with Mental Disorders. Proceedings of the Annual Conference of the Japanese Academy of Psychiatric and Mental Health Nursing. 2019;29:93 (In Japanese).
184. Nakamura H, Watanabe N. Factors Influencing the Resilience of Individuals with Schizophrenia Living in the Community. Proceedings of the Annual Conference of the Japanese Academy of Psychiatric and Mental Health Nursing. 2019;29:149 (In Japanese).
185. Ministry of Health, Labour and Welfare. White Paper on Health, Labour and Welfare 2023 [Internet]. Ministry of Health, Labour and Welfare. 2023 [cited 2025 Oct 23] (In Japanese). Available from: https://www.mhlw.go.jp/stf/wp/hakusyo/kousei/22/
186. The National Federation of Associations of Families with The Mental Illness in Japan (Common name: Minna-net). Development and Implementation of the Japanese Schizophrenia Caregiver Questionnaire (J-SCQ) (Naotoshi Kanzawa) [Internet]. The National Federation of Associations of Families with The Mental Illness in Japan. 2023 [cited 2025 Oct 23] (In Japanese). Available from: https://seishinhoken.jp/researches/01gpf46v587ysjag0nh60a2egh
187. Ministry of Health, Labour and Welfare. Survey on Medical Insurance Benefits FY 2021 [Internet]. Government Statistics Service (e-Stat). 2023 [cited 2025 Oct 23] (In Japanese). Available from: https://www.e-stat.go.jp/stat-search/files?page=1&layout=datalist&toukei=00450389&tstat=000001044924&cycle=0&tclass1=000001044945&tclass2=000001209743&tclass3val=0
188. Ministry of Health, Labour and Welfare. Patient Survey 2020: Table 3 (Estimated Number of Patients by Place of Residence, Type of Facility, Inpatient-Outpatient, Within and Outside Prefecture, by Prefecture; Estimated Number and Proportion of Outflow Patients, Inpatient-Outpatient, by Prefecture) [Internet]. Government Statistics Service (e-Stat). 2022 [cited 2025 Oct 23] (In Japanese). Available from: https://www.e-stat.go.jp/dbview?sid=0004002833
189. Ministry of Health, Labour and Welfare. Patient Survey 2020: Table 4 (Estimated Number of Inpatients by Location of Medical Facility, Type of Facility, Inpatient–Outpatient, Within and Outside Prefecture, by Prefecture; by Disease Classification) [Internet]. Government Statistics Service (e-Stat). 2022 [cited 2025 Oct 23] (In Japanese). Available from: https://www.e-stat.go.jp/dbview?sid=0004002834
190. Ministry of Health, Labour and Welfare. Patient Survey 2020: Table 4 (Estimated Number of Outpatients by Location of Medical Facility, Type of Facility, Inpatient–Outpatient, Within and Outside Prefecture, by Prefecture; by Disease Classification) [Internet]. Government Statistics Service (e-Stat). 2022 [cited 2025 Oct 23] (In Japanese). Available from: https://www.e-stat.go.jp/dbview?sid=0004002834
191. Ministry of Health, Labour and Welfare. Patient Survey 2020: Table 5 (Estimated Number of Patients by Place of Residence, Type of Facility, Inpatient–Outpatient, Within and Outside Prefecture, by Prefecture; by Disease Classification) [Internet]. Government Statistics Service (e-Stat). 2022 [cited 2025 Oct 23] (In Japanese). Available from: https://www.e-stat.go.jp/dbview?sid=0004002835
192. Ministry of Health, Labour and Welfare. Overview of the Social Medical Care Fee Statistics FY2022 [Internet]. Ministry of Health, Labour and Welfare. 2022 [cited 2025 Oct 23] (In Japanese). Available from: https://www.mhlw.go.jp/toukei/saikin/hw/sinryo/tyosa22/
193. Ministry of Health, Labour and Welfare. White Paper on Health, Labour and Welfare 2022 [Internet]. Ministry of Health, Labour and Welfare. 2022 [cited 2025 Oct 23] (In Japanese). Available from: https://www.mhlw.go.jp/stf/wp/hakusyo/kousei/21/
194. Ministry of Health, Labour and Welfare. Survey of Home-Visit Nursing Care Expenses FY 2021 [Internet]. Government Statistics Service (e-Stat). 2022 [cited 2025 Oct 23] (In Japanese). Available from: https://www.e-stat.go.jp/stat-search/files?page=1&layout=datalist&toukei=00450385&tstat=000001052926&cycle=0&tclass1=000001163886&tclass2=000001220362&tclass3val=0
195. Ministry of Health, Labour and Welfare. Patient Survey 2020: Table 7 (Treatment Rate of Inpatients (per 100,000 Population) by Type of Facility, Inpatient–Outpatient, by Disease Classification and Prefecture) [Internet]. Government Statistics Service (e-Stat). 2022 [cited 2025 Oct 23] (In Japanese). Available from: https://www.e-stat.go.jp/dbview?sid=0004002837
196. Ministry of Health, Labour and Welfare. Patient Survey 2020: Table 7 (Treatment Rate of Outpatients (per 100,000 Population) by Type of Facility, Inpatient–Outpatient, by Disease Classification and Prefecture) [Internet]. Government Statistics Service (e-Stat). 2022 [cited 2025 Oct 23] (In Japanese). Available from: https://www.e-stat.go.jp/dbview?sid=0004002837
197. Ministry of Health, Labour and Welfare. Survey on Medical Insurance Benefits FY 2020 [Internet]. Government Statistics Service (e-Stat). 2022 [cited 2025 Oct 23] (In Japanese). Available from: https://www.e-stat.go.jp/stat-search/files?page=1&layout=datalist&toukei=00450389&tstat=000001044924&cycle=0&tclass1=000001044945&tclass2=000001171146&tclass3val=0
198. Ministry of Health, Labour and Welfare. Survey on the Evaluation of the Impact of DPC Implementation FY 2019: Reference material 2 (9) [Internet]. Ministry of Health, Labour and Welfare. 2021 [cited 2025 Oct 23] (In Japanese). Available from: https://view.officeapps.live.com/op/view.aspx?src=https%3A%2F%2Fwww.mhlw.go.jp%2Fcontent%2F12404000%2F001350532.xlsx&wdOrigin=BROWSELINK
199. Ministry of Health, Labour and Welfare. Survey on the Evaluation of the Impact of DPC Implementation FY 2019: Reference material 2 (10) [Internet]. Ministry of Health, Labour and Welfare. 2021 [cited 2025 Oct 23] (In Japanese). Available from: https://view.officeapps.live.com/op/view.aspx?src=https%3A%2F%2Fwww.mhlw.go.jp%2Fcontent%2F12404000%2F001350576.xlsx&wdOrigin=BROWSELINK
200. Ministry of Health, Labour and Welfare. Survey on the Evaluation of the Impact of DPC Implementation FY 2019: Reference material 1 (13) [Internet]. Ministry of Health, Labour and Welfare. 2021 [cited 2025 Oct 23] (In Japanese). Available from: https://view.officeapps.live.com/op/view.aspx?src=https%3A%2F%2Fwww.mhlw.go.jp%2Fcontent%2F12404000%2F001350418.xlsx&wdOrigin=BROWSELINK
201. Ministry of Health, Labour and Welfare. Survey on the Evaluation of the Impact of DPC Implementation FY 2019: Reference material 2 (6) [Internet]. Ministry of Health, Labour and Welfare. 2021 [cited 2025 Oct 23] (In Japanese). Available from: https://view.officeapps.live.com/op/view.aspx?src=https%3A%2F%2Fwww.mhlw.go.jp%2Fcontent%2F12404000%2F001350440.xlsx&wdOrigin=BROWSELINK
202. Ministry of Health, Labour and Welfare. Survey on the Evaluation of the Impact of DPC Implementation FY 2019: Reference material 1 (16) [Internet]. Ministry of Health, Labour and Welfare. 2021 [cited 2025 Oct 23] (In Japanese). Available from: https://view.officeapps.live.com/op/view.aspx?src=https%3A%2F%2Fwww.mhlw.go.jp%2Fcontent%2F12404000%2F001350421.xlsx&wdOrigin=BROWSELINK
203. Ministry of Health, Labour and Welfare. Overview of the Social Medical Care Fee Statistics FY2021 [Internet]. Ministry of Health, Labour and Welfare. 2021 [cited 2025 Oct 23] (In Japanese). Available from: https://www.mhlw.go.jp/toukei/saikin/hw/sinryo/tyosa21/
204. Ministry of Health, Labour and Welfare. White Paper on Health, Labour and Welfare 2021 [Internet]. Ministry of Health, Labour and Welfare. 2021 [cited 2025 Oct 23] (In Japanese). Available from: https://www.mhlw.go.jp/stf/wp/hakusyo/kousei/20/
205. Ministry of Health, Labour and Welfare. Overview of National Medical Care Expenditures FY2021 [Internet]. Ministry of Health, Labour and Welfare. 2021 [cited 2025 Oct 23] (In Japanese). Available from: https://www.mhlw.go.jp/toukei/saikin/hw/k-iryohi/21/index.html
206. Ministry of Health, Labour and Welfare. Survey on Medical Insurance Benefits FY 2019 [Internet]. Government Statistics Service (e-Stat). 2021 [cited 2025 Oct 23] (In Japanese). Available from: https://www.e-stat.go.jp/stat-search/files?page=1&layout=datalist&toukei=00450389&tstat=000001044924&cycle=0&tclass1=000001044945&tclass2=000001156339&tclass3val=0
207. Ministry of Health, Labour and Welfare. Summary of Patient Survey 2020 [Internet]. Ministry of Health, Labour and Welfare. 2020 [cited 2025 Oct 23] (In Japanese). Available from: https://www.mhlw.go.jp/toukei/saikin/hw/kanja/20/index.html
208. Ministry of Health, Labour and Welfare. Survey on the Evaluation of the Impact of DPC Implementation FY 2018: Reference material 2 (9) [Internet]. Ministry of Health, Labour and Welfare. 2020 [cited 2025 Oct 23] (In Japanese). Available from: https://view.officeapps.live.com/op/view.aspx?src=https%3A%2F%2Fwww.mhlw.go.jp%2Fcontent%2F12404000%2F001351861.xlsx&wdOrigin=BROWSELINK
209. Ministry of Health, Labour and Welfare. Survey on the Evaluation of the Impact of DPC Implementation FY 2018: Reference material 2 (10) [Internet]. Ministry of Health, Labour and Welfare. 2020 [cited 2025 Oct 23] (In Japanese). Available from: https://view.officeapps.live.com/op/view.aspx?src=https%3A%2F%2Fwww.mhlw.go.jp%2Fcontent%2F12404000%2F001351931.xlsx&wdOrigin=BROWSELINK
210. Ministry of Health, Labour and Welfare. Survey on the Evaluation of the Impact of DPC Implementation FY 2018: Reference material 1 (13) [Internet]. Ministry of Health, Labour and Welfare. 2020 [cited 2025 Oct 23] (In Japanese). Available from: https://view.officeapps.live.com/op/view.aspx?src=https%3A%2F%2Fwww.mhlw.go.jp%2Fcontent%2F12404000%2F001351756.xlsx&wdOrigin=BROWSELINK
211. Ministry of Health, Labour and Welfare. Survey on the Evaluation of the Impact of DPC Implementation FY 2018: Reference material 2 (6) [Internet]. Ministry of Health, Labour and Welfare. 2020 [cited 2025 Oct 23] (In Japanese). Available from: https://view.officeapps.live.com/op/view.aspx?src=https%3A%2F%2Fwww.mhlw.go.jp%2Fcontent%2F12404000%2F001351782.xlsx&wdOrigin=BROWSELINK
212. Ministry of Health, Labour and Welfare. Survey on the Evaluation of the Impact of DPC Implementation FY 2018: Reference material 1 (16) [Internet]. Ministry of Health, Labour and Welfare. 2020 [cited 2025 Oct 23] (In Japanese). Available from: https://view.officeapps.live.com/op/view.aspx?src=https%3A%2F%2Fwww.mhlw.go.jp%2Fcontent%2F12404000%2F001351761.xlsx&wdOrigin=BROWSELINK
213. Ministry of Health, Labour and Welfare. Overview of the Social Medical Care Fee Statistics FY2020 [Internet]. Ministry of Health, Labour and Welfare. 2020 [cited 2025 Oct 23] (In Japanese). Available from: https://www.mhlw.go.jp/toukei/saikin/hw/sinryo/tyosa20/
214. Ministry of Health, Labour and Welfare. White Paper on Health, Labour and Welfare 2020 [Internet]. Ministry of Health, Labour and Welfare. 2020 [cited 2025 Oct 23] (In Japanese). Available from: https://www.mhlw.go.jp/stf/wp/hakusyo/kousei/19/
215. Ministry of Health, Labour and Welfare. Survey of Home-Visit Nursing Care Expenses FY 2019 [Internet]. Government Statistics Service (e-Stat). 2020 [cited 2025 Oct 23] (In Japanese). Available from: https://www.e-stat.go.jp/stat-search/files?page=1&layout=datalist&toukei=00450385&tstat=000001052926&cycle=0&tclass1=000001137746&tclass2val=0
216. Ministry of Health, Labour and Welfare. Overview of National Medical Care Expenditures FY2020 [Internet]. Ministry of Health, Labour and Welfare. 2020 [cited 2025 Oct 23] (In Japanese). Available from: https://www.mhlw.go.jp/toukei/saikin/hw/k-iryohi/20/index.html
217. Ministry of Health, Labour and Welfare. Survey on Medical Insurance Benefits FY 2018 [Internet]. Government Statistics Service (e-Stat). 2020 [cited 2025 Oct 23] (In Japanese). Available from: https://www.e-stat.go.jp/stat-search/files?page=1&layout=datalist&toukei=00450389&tstat=000001044924&cycle=0&tclass1=000001044945&tclass2=000001143146&tclass3val=0
218. Ministry of Health, Labour and Welfare. Overview of the Social Medical Care Fee Statistics FY2019 [Internet]. Ministry of Health, Labour and Welfare. 2019 [cited 2025 Oct 23] (In Japanese). Available from: https://www.mhlw.go.jp/toukei/saikin/hw/sinryo/tyosa19/
219. Ministry of Health, Labour and Welfare. Overview of National Medical Care Expenditures FY2019 [Internet]. Ministry of Health, Labour and Welfare. 2019 [cited 2025 Oct 23] (In Japanese). Available from: https://www.mhlw.go.jp/toukei/saikin/hw/k-iryohi/19/index.html
220. Ministry of Health, Labour and Welfare. Overview of the Social Medical Care Fee Statistics FY2018 [Internet]. Ministry of Health, Labour and Welfare. 2018 [cited 2025 Oct 23] (In Japanese). Available from: https://www.mhlw.go.jp/toukei/saikin/hw/sinryo/tyosa18/
221. Ministry of Health, Labour and Welfare. Overview of National Medical Care Expenditures FY2018 [Internet]. Ministry of Health, Labour and Welfare. 2018 [cited 2025 Oct 23] (In Japanese). Available from: https://www.mhlw.go.jp/toukei/saikin/hw/k-iryohi/18/index.html
